# Supplementary material for: Effect of Hydroxyl Groups Esterification with Fatty Acids on the Cytotoxicity and Antioxidant Activity of Flavones
Source: Molecules. 2022 Jan 10;27(2):420. doi: 10.3390/molecules27020420 (PMC8777613; doi:10.3390/molecules27020420)
Supplement: Supplementary file 1 [file molecules-27-00420-s001.zip › molecules-1499940-supplementary.pdf]

Supplementary Materials

Article

Effect of hydroxyl groups esterification with fatty acids on the cytotoxicity and antioxidant activity of flavones.

Grażyna Kubiak-Tomaszewska[1], Piotr Roszkowski[2,*], Emilia Grosicka-Maciąg[3,*] , Paulina Strzyga-Łach[3] **and** Marta Struga[3]

[1] Department of Biochemistry and Pharmacogenomics, Faculty of Pharmacy, Medical University of Warsaw, Banacha 1, 02-097 Warszawa; grazyna.kubiak-tomaszewska@wum.edu.pl

[2] Faculty of Chemistry, University of Warsaw, Pasteura 1, 02-093 Warsaw, Poland; roszkowski@chem.uw.edu.pl

[3] Chair and Department of Biochemistry, Medical University of Warsaw, Banacha 1, 02-097 Warszawa, Poland ; (M.S) marta.struga@wum.edu.pl, (E.G-M) emilia.grosicka-maciag@wum.edu.pl, (P.S-Ł) paulina.strzyga@wum.edu.pl

* Correspondence: chemistry studies: (P.R) roszkowski@chem.uw.edu.pl; biological studies: (E.G-M) emilia.grosicka-maciag@wum.edu.pl

Table S1. Cells viability (%). Representative MTT assay results of the studied compounds towards prostate cancer cells (PC3) - **A** and human keratinocyte cells (HaCaT) - **B** after 72 h exposure. **1** – 6-Hydroxy-Flavanone, **2** – 6-Stearic-Flavanone, **3** – 6-Oleic-Flavanone, **4** – 6-Sorbic-Flavanone, **5** – 6-Linolenic-Flavanone, **6** – 6-Linoleic-Flavanone, **7** – 7-Hydroxy-Flavanone, **8** – 7-Stearic-Flavanone, **9** – 7-Oleic-Flavanone, **10** – 7-Sorbic-Flavanone, **11** – 7-Linolenic-Flavanone, **12** – 7-Linoleic-Flavanone, **13** – 6-Hydroxy-Flavone, **14** – 7-Hydroksy-Flavone.

A

**Compound**	**1**	**2**	**3**	**4**	**5**	**6**	**7**	**8**	**9**	**10**	**11**	**12**	**13**	**14**
**Concentration (µM)**														
**10**	96.42	96.55	100	95.67	92.59	94.72	79.77	100	92.22	84	90.28	84.66	89.46	123
**20**	96.01	106	70.93	88.72	85.90	76.52	83.98	87.73	80.88	80.99	86.39	70.45	87.88	117
**40**	96.02	99.61	41.45	82.18	79.53	53.31	79.39	41.83	48.23	93.62	81.72	46.70	99.04	111
**60**	96.70	93.95	19.37	41.35	48.08	45.46	68.98	32.23	41.06	74.54	79.46	35.98	110	95.45
**80**	96.45	93.62	13.32	28.73	35.87	31.26	76.69	47.90	42.20	69.67	86.88	34.33	87.06	65.03
**100**	96.80	93.74	6.81	32.64	31.58	20.25	70.98	27.58	38.92	75.20	86.76	39.50	73.27	62.78

B

**Compound**	**1**	**2**	**3**	**4**	**5**	**6**	**7**	**8**	**9**	**10**	**11**	**12**	**13**	**14**
**Concentration (µM)**														
**10**	99.65	95.43	97.81	98.70	95.83	95.69	97.17	95.81	95.35	97.45	106	88.37	96.40	93.76
**20**	115	82.54	69.96	96.71	75.24	93.76	102	65.27	79.35	87.21	90.54	73.18	83.83	65.84
**40**	113	54.71	75.66	89.56	53.27	86.83	96.70	59.07	51.85	80.07	66.03	74.45	77.90	69.65
**60**	104	47.76	26.37	75.70	38.07	73.39	84.03	39.84	39.87	40.95	19.41	57.01	95.12	96.17
**80**	72.15	32.51	26.52	56.71	25.78	50.27	93.41	35.66	38.67	57.37	15.80	44.63	84.89	97.17
**100**	74.44	11.67	26.50	31.37	23.79	34.88	86.46	20.72	44.93	43.68	14.63	34.01	87.84	92.38

Figure S1: Supporting NMR spectra of synthesized esters of 6-hydroxy-flavanone and 7-hydroxy-flavone

The ^{1}H and ^{13}C NMR spectra are presented in the following order:

6-hydroxyflavanone sorbic ester (**4a**)

7-hydroxyflavone sorbic ester (**5a**)

6-hydroxyflavanone stearic ester (**4b**)

7-hydroxyflavone stearic ester (**5b**)

6-hydroxyflavanone oleic ester (**4c**)

7-hydroxyflavone oleic ester (**5c**)

6-hydroxyflavanone linoleic ester (**4d**)

7-hydroxyflavone linoleic ester (**5d**)

6-hydroxyflavanone linolenic ester (**4e**)

7-hydroxyflavone linolenic ester (**5e**)

The selected MS spectra are presented in the following order:

6-hydroxyflavanone sorbic ester (**4a**)

7-hydroxyflavone sorbic ester (**5a**)

6-hydroxyflavanone oleic ester (**4c**)

7-hydroxyflavone oleic ester (**5c**)

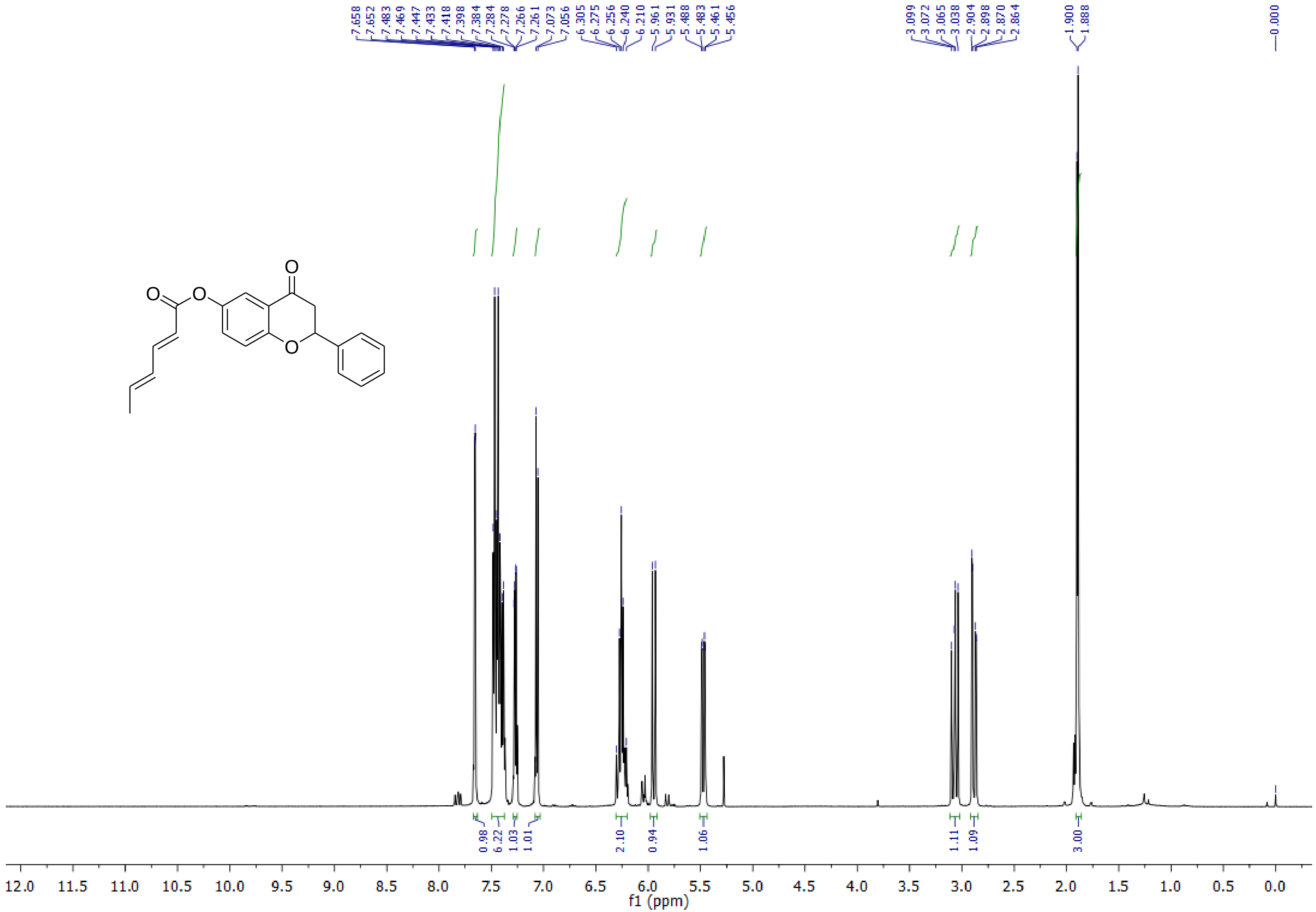
7.658
7.652
7.483
7.469
7.447
7.433
7.418
7.398
7.384
7.284
7.278
7.266
7.261
7.073
7.056
6.305
6.275
6.256
6.240
6.210
5.961
5.931
5.488
5.483
5.461
5.456
3.099
3.072
3.065
3.038
2.904
2.898
2.870
2.864
1.900
1.888
0.000
0.98
6.22
1.03
1.01
2.10
0.94
1.06
1.11
1.09
3.00
12.0
11.5
11.0
10.5
10.0
9.5
9.0
8.5
8.0
7.5
7.0
6.5
6.0
5.5
5.0
4.5
4.0
3.5
3.0
2.5
2.0
1.5
1.0
0.5
0.0
f1 (ppm)

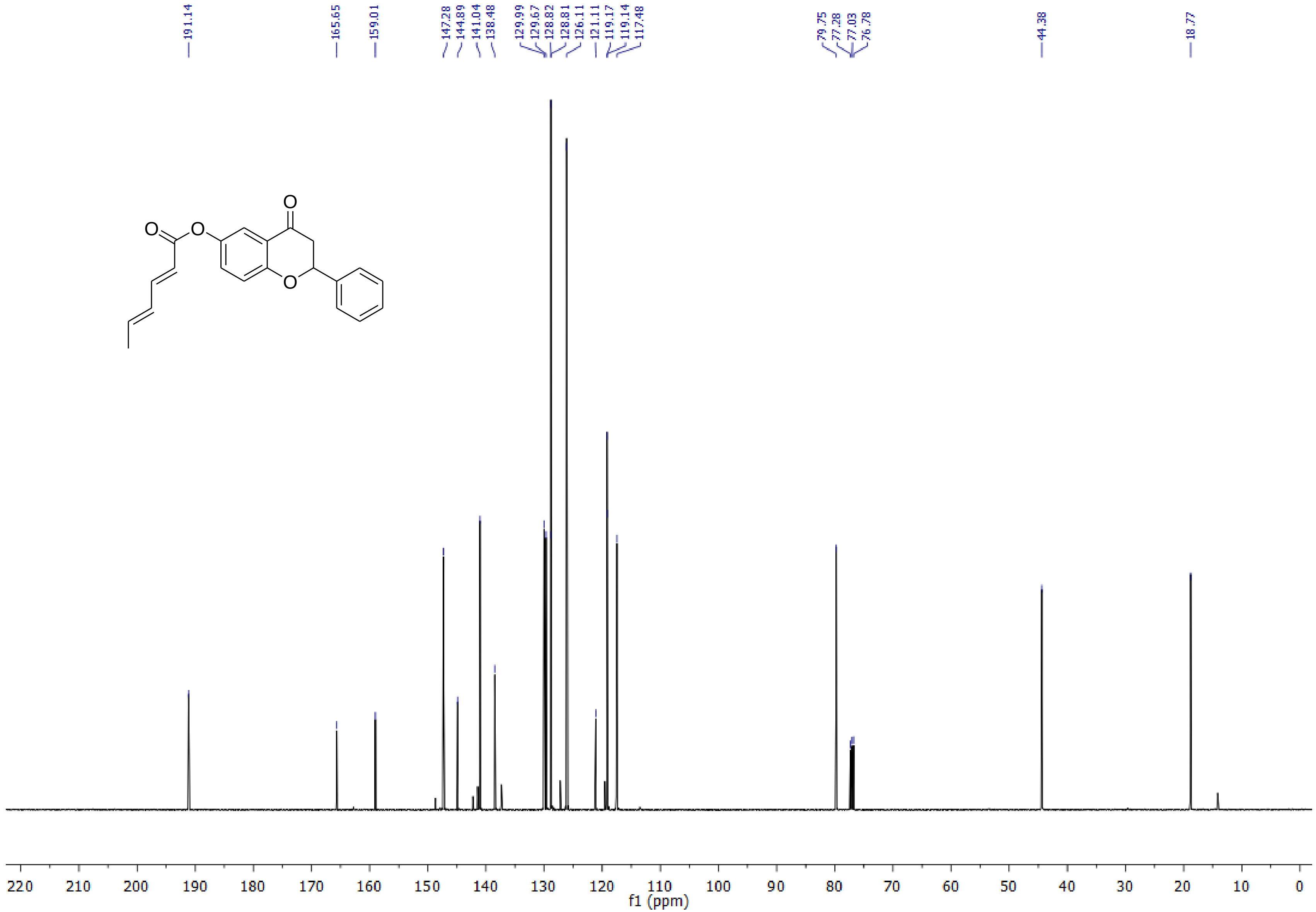
191.14
165.65
159.01
147.28
144.89
141.04
138.48
129.99
129.67
128.82
128.81
126.11
121.11
119.17
119.14
117.48
79.75
77.28
77.03
76.78
44.38
18.77
220 210 200 190 180 170 160 150 140 130 120 110 100 90 80 70 60 50 40 30 20 10 0
f1 (ppm)

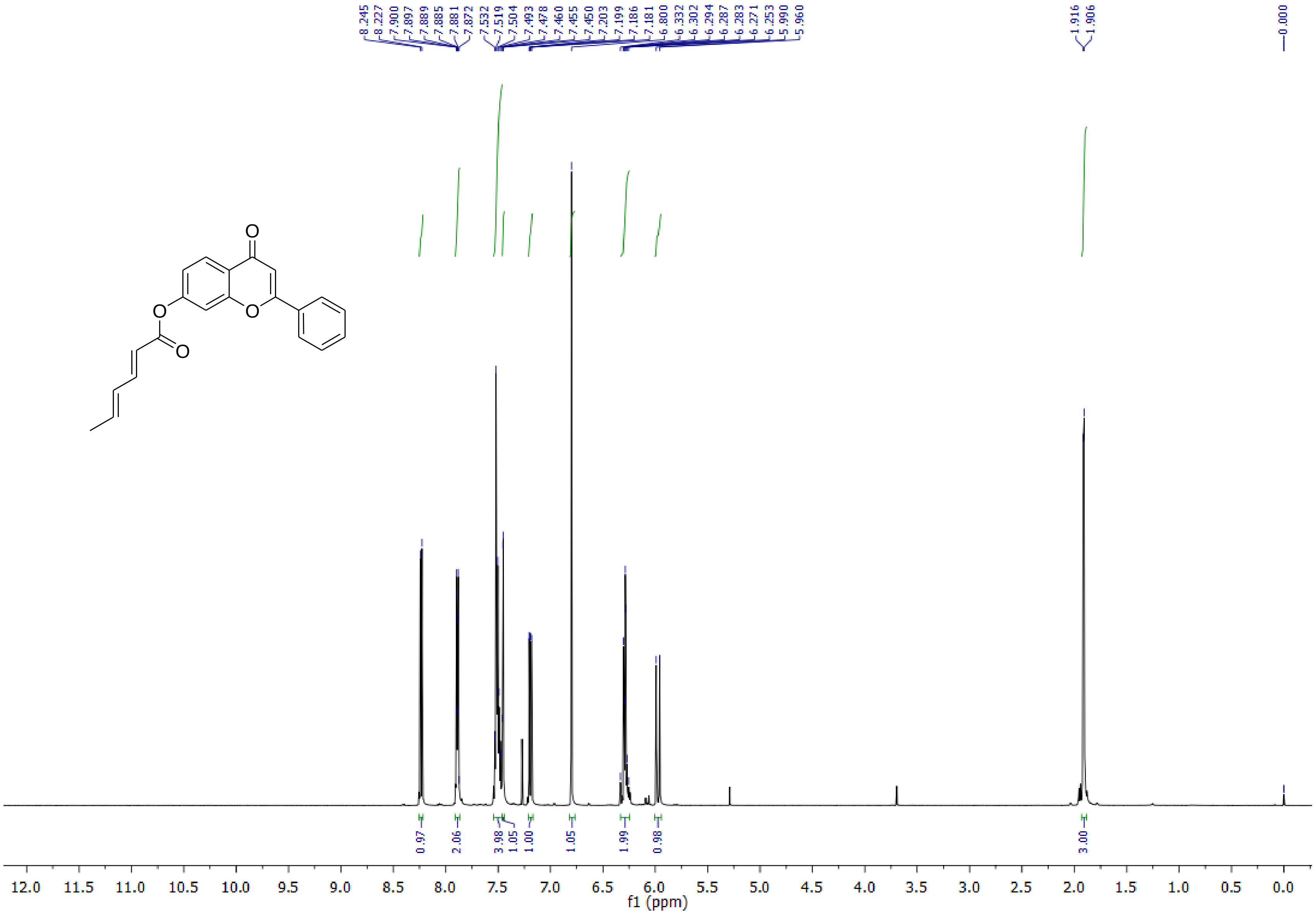
8.245
8.227
7.900
7.897
7.889
7.885
7.881
7.872
7.532
7.519
7.504
7.493
7.478
7.460
7.455
7.450
7.203
7.199
7.186
7.181
6.800
6.332
6.302
6.294
6.287
6.283
6.271
6.253
5.990
5.960
1.916
1.906
0.000
0.97
2.06
3.98
1.05
1.00
1.05
1.99
0.98
3.00
12.0 11.5 11.0 10.5 10.0 9.5 9.0 8.5 8.0 7.5 7.0 6.5 6.0 5.5 5.0 4.5 4.0 3.5 3.0 2.5 2.0 1.5 1.0 0.5 0.0
f1 (ppm)
O
O
O
O

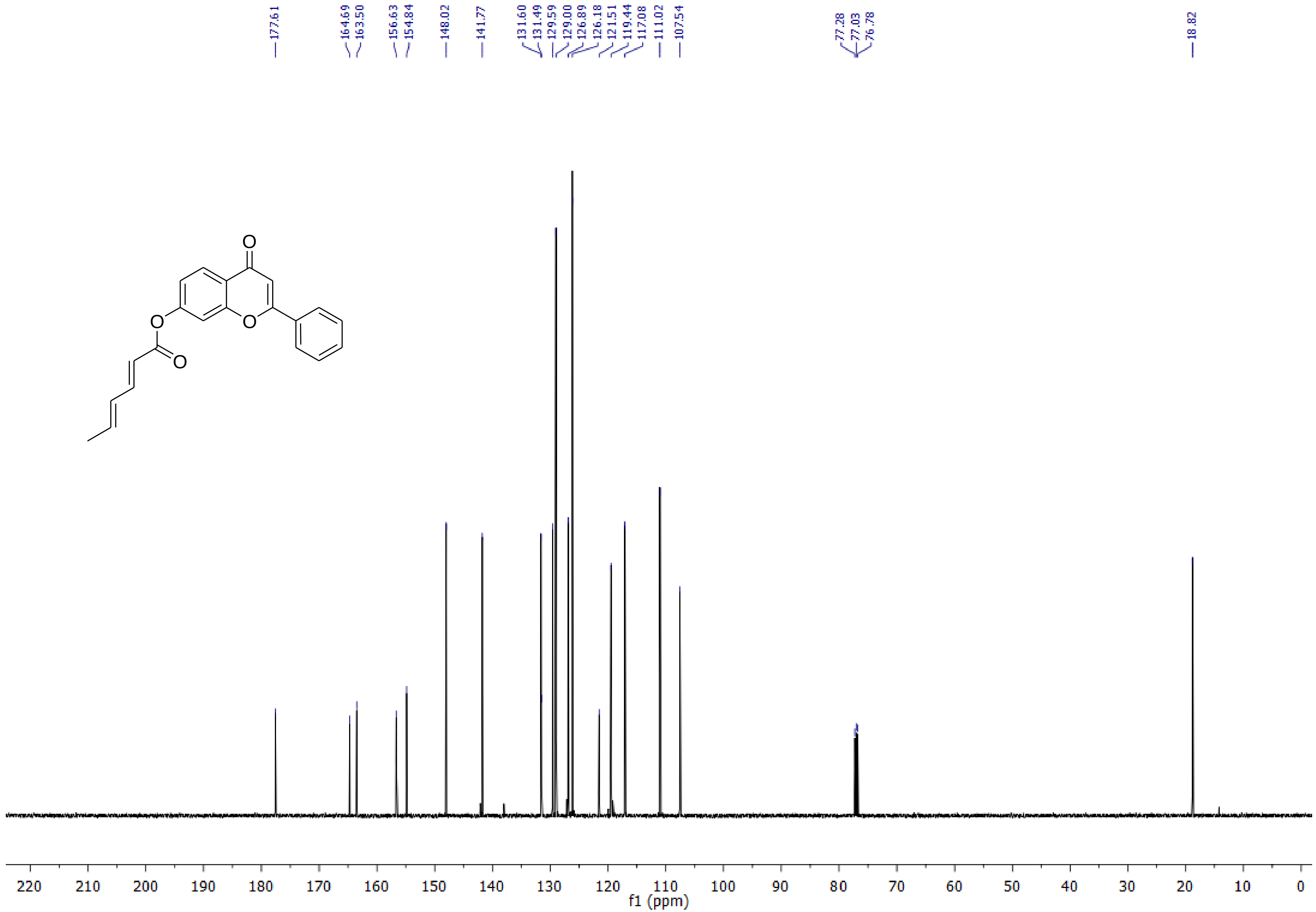
177.61
164.69
163.50
156.63
154.84
148.02
141.77
131.60
131.49
129.59
129.00
126.89
126.18
121.51
119.44
117.08
111.02
107.54
77.28
77.03
76.78
18.82
O
O
O
O
220
210
200
190
180
170
160
150
140
130
120
110
100
90
80
70
60
50
40
30
20
10
0
f1 (ppm)

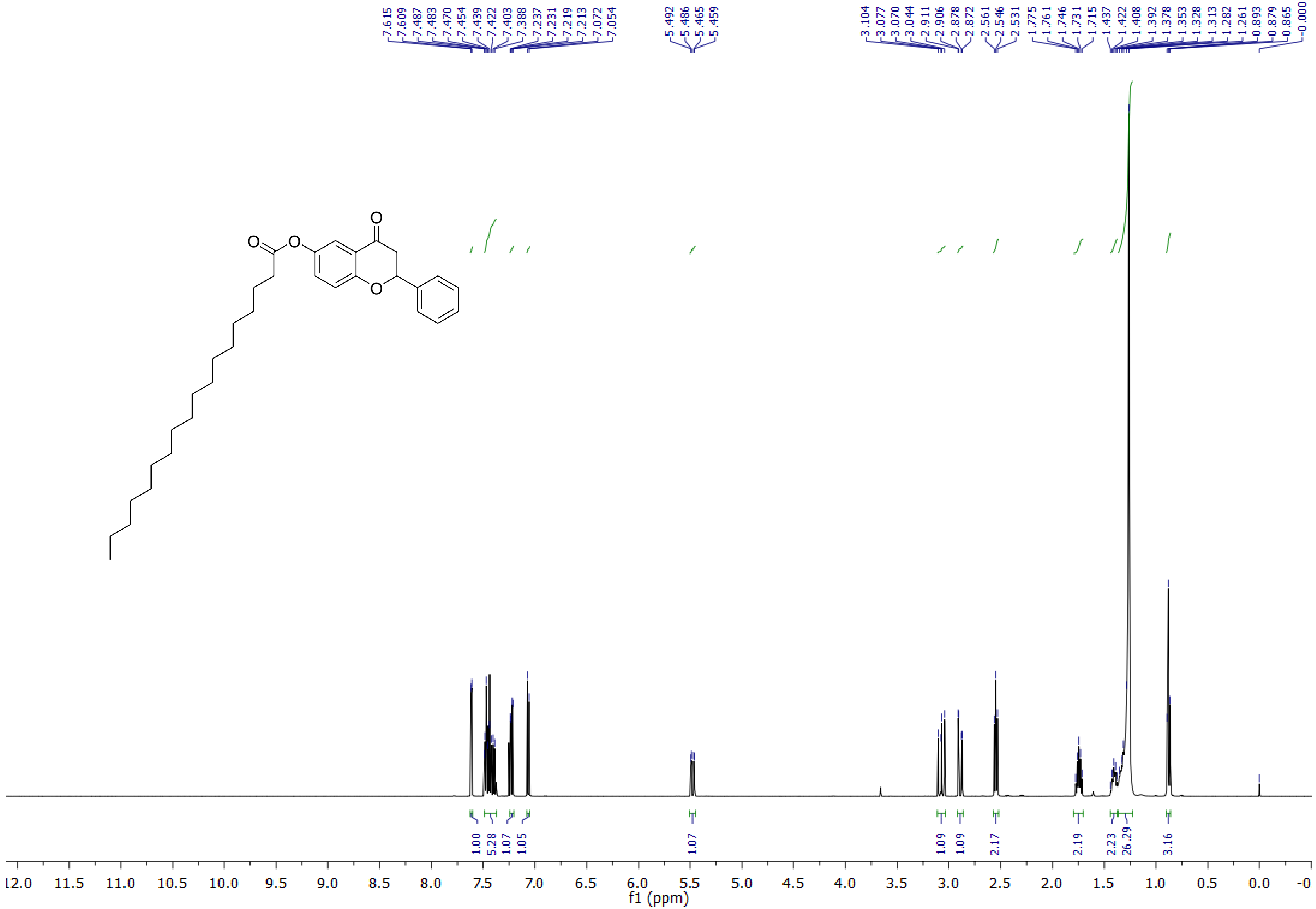

7.615 7.609 7.487 7.483 7.470 7.454 7.439 7.422 7.403 7.388 7.237 7.231 7.219 7.213 7.072 7.054
5.492 5.486 5.465 5.459
3.104 3.077 3.070 3.044 2.911 2.906 2.878 2.872 2.561 2.546 2.531 1.775 1.761 1.746 1.731 1.715 1.437 1.422 1.408 1.392 1.378 1.353 1.328 1.313 1.282 1.261 0.893 0.879 0.865 -0.000
1.00 5.28 1.07 1.05 1.07 1.09 1.09 2.17 2.19 2.23 26.29 3.16
f1 (ppm)

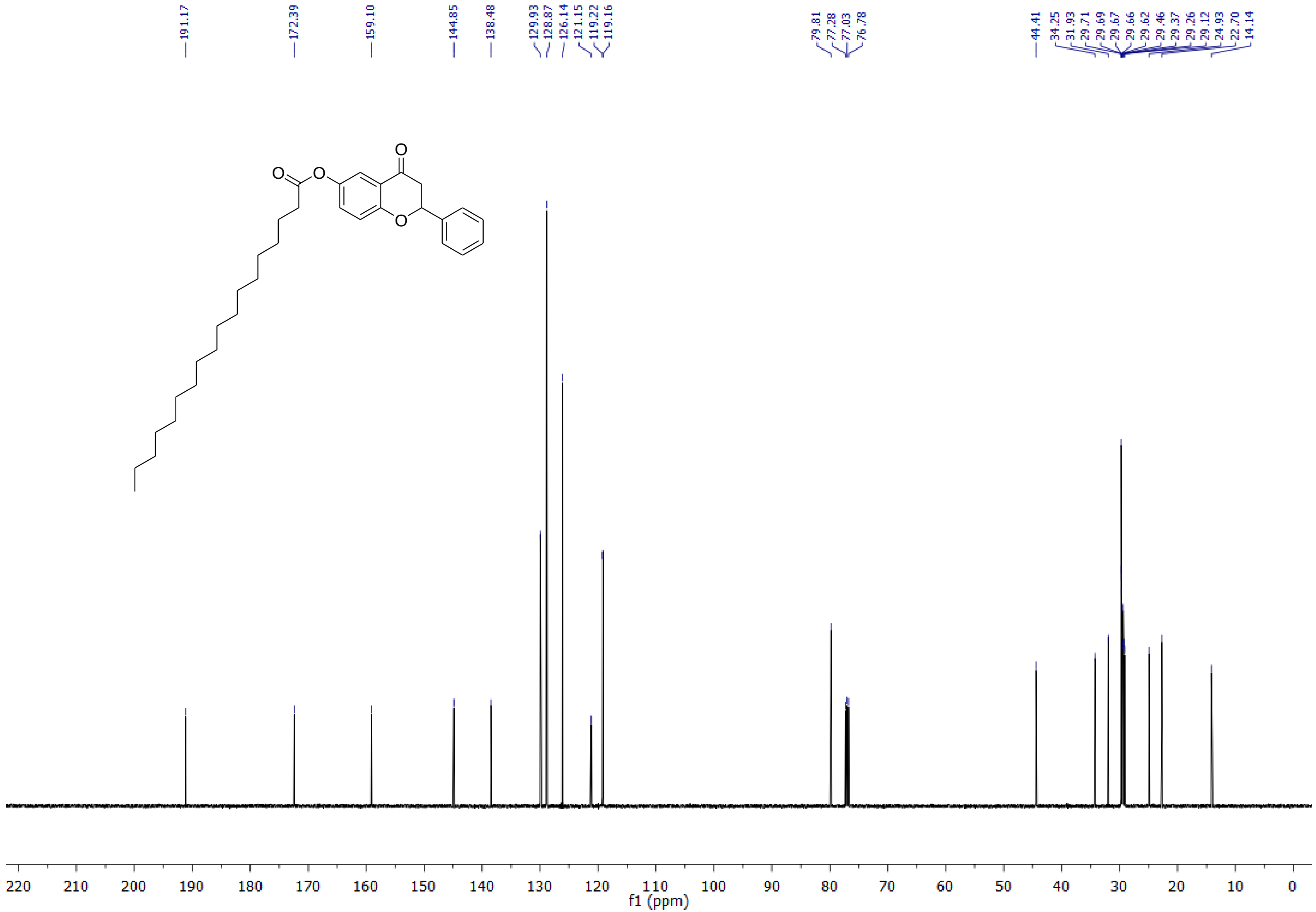
191.17
172.39
159.10
144.85
138.48
129.93
128.87
126.14
121.15
119.22
119.16
79.81
77.28
77.03
76.78
44.41
34.25
31.93
29.71
29.69
29.67
29.66
29.62
29.46
29.37
29.26
29.12
24.93
22.70
14.14
O
O
O
O
220 210 200 190 180 170 160 150 140 130 120 110 100 90 80 70 60 50 40 30 20 10 0
f1 (ppm)

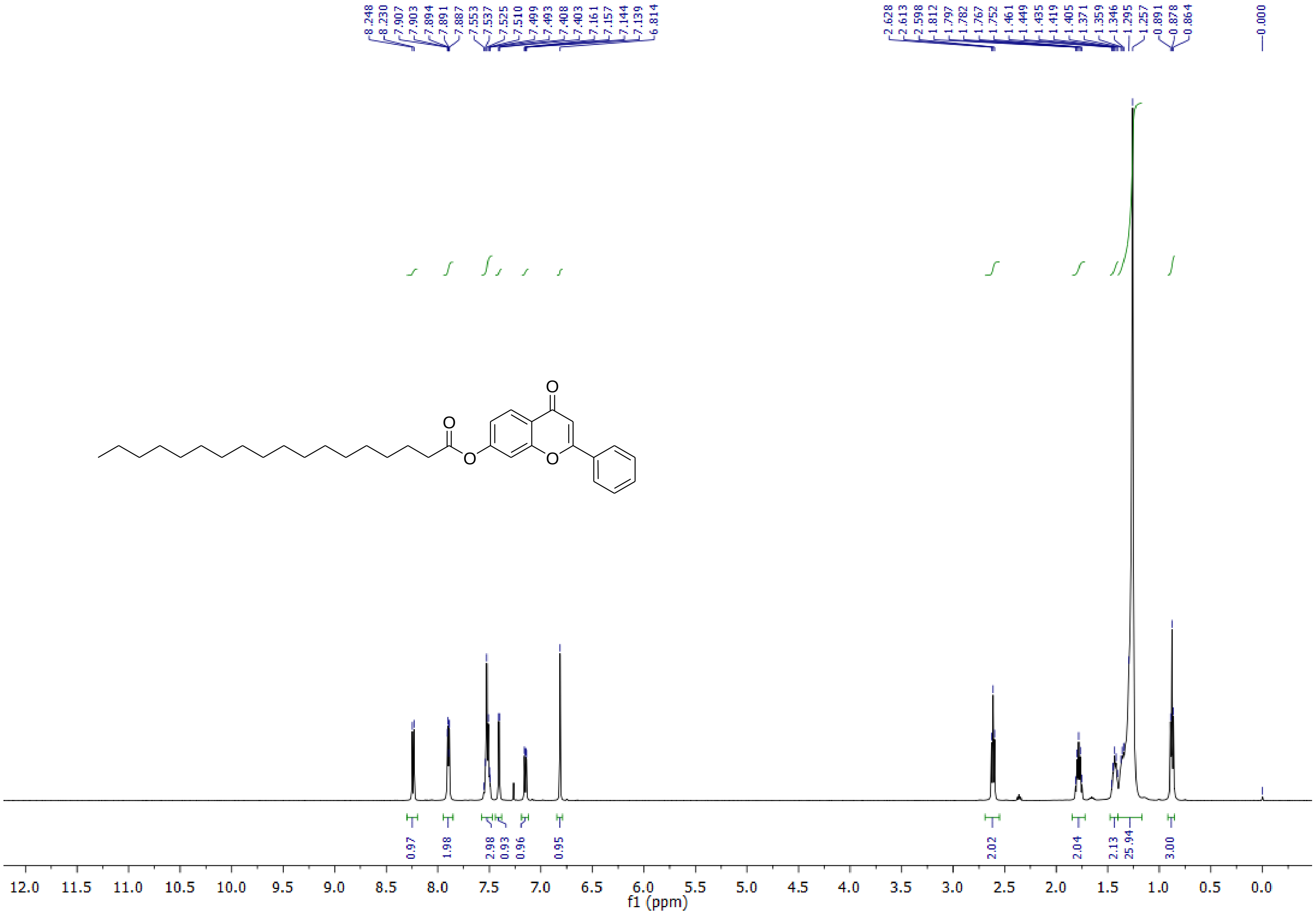

8.248
8.230
7.907
7.903
7.894
7.891
7.887
7.553
7.537
7.525
7.510
7.499
7.493
7.408
7.403
7.161
7.157
7.144
7.139
6.814
2.628
2.613
2.598
1.812
1.797
1.782
1.767
1.752
1.461
1.449
1.435
1.419
1.405
1.371
1.359
1.346
1.295
1.257
0.891
0.878
0.864
0.000
0.97
1.98
2.98
0.93
0.96
0.95
2.02
2.04
2.13
25.94
3.00
12.0 11.5 11.0 10.5 10.0 9.5 9.0 8.5 8.0 7.5 7.0 6.5 6.0 5.5 5.0 4.5 4.0 3.5 3.0 2.5 2.0 1.5 1.0 0.5 0.0
f1 (ppm)

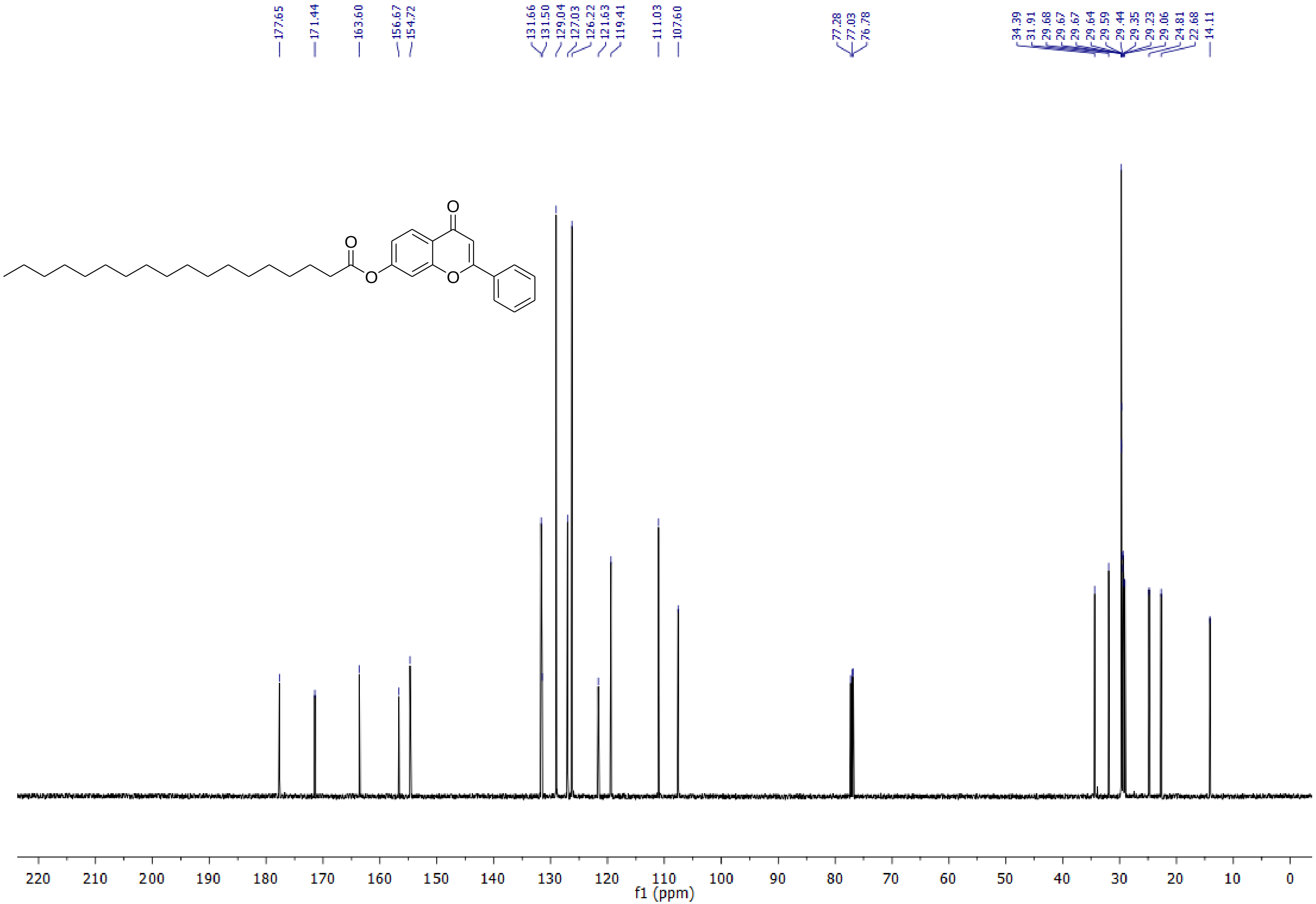
177.65
171.44
163.60
156.67
154.72
131.66
131.50
129.04
127.03
126.22
121.63
119.41
111.03
107.60
77.28
77.03
76.78
34.39
31.91
29.68
29.67
29.67
29.64
29.59
29.44
29.35
29.23
29.06
24.81
22.68
14.11
220 210 200 190 180 170 160 150 140 130 120 110 100 90 80 70 60 50 40 30 20 10 0
f1 (ppm)

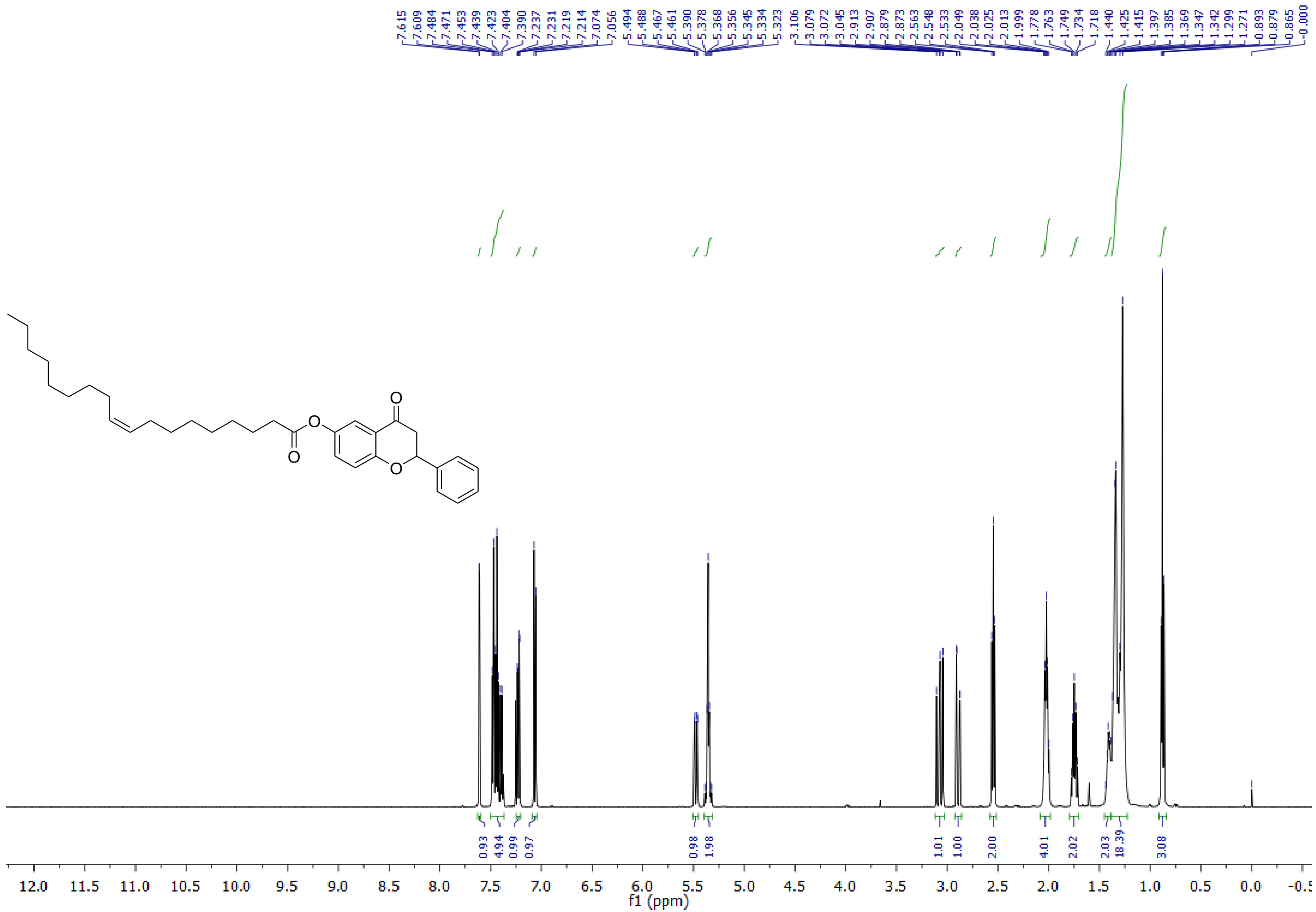
7.615 7.609 7.484 7.471 7.453 7.439 7.423 7.404 7.390 7.237 7.231 7.219 7.214 7.074 7.056 5.494 5.488 5.467 5.461 5.390 5.378 5.368 5.356 5.345 5.334 5.323 3.106 3.079 3.072 3.045 2.913 2.907 2.879 2.873 2.563 2.548 2.533 2.049 2.038 2.025 2.013 1.999 1.778 1.763 1.749 1.734 1.718 1.440 1.425 1.415 1.397 1.385 1.369 1.347 1.342 1.299 1.271 0.893 0.879 0.865 -0.000
0.93 4.94 0.99 0.97 0.98 1.98 1.01 1.00 2.00 4.01 2.02 2.03 18.39 3.08
12.0 11.5 11.0 10.5 10.0 9.5 9.0 8.5 8.0 7.5 7.0 6.5 6.0 5.5 5.0 4.5 4.0 3.5 3.0 2.5 2.0 1.5 1.0 0.5 0.0
f1 (ppm)
O
O
O
O

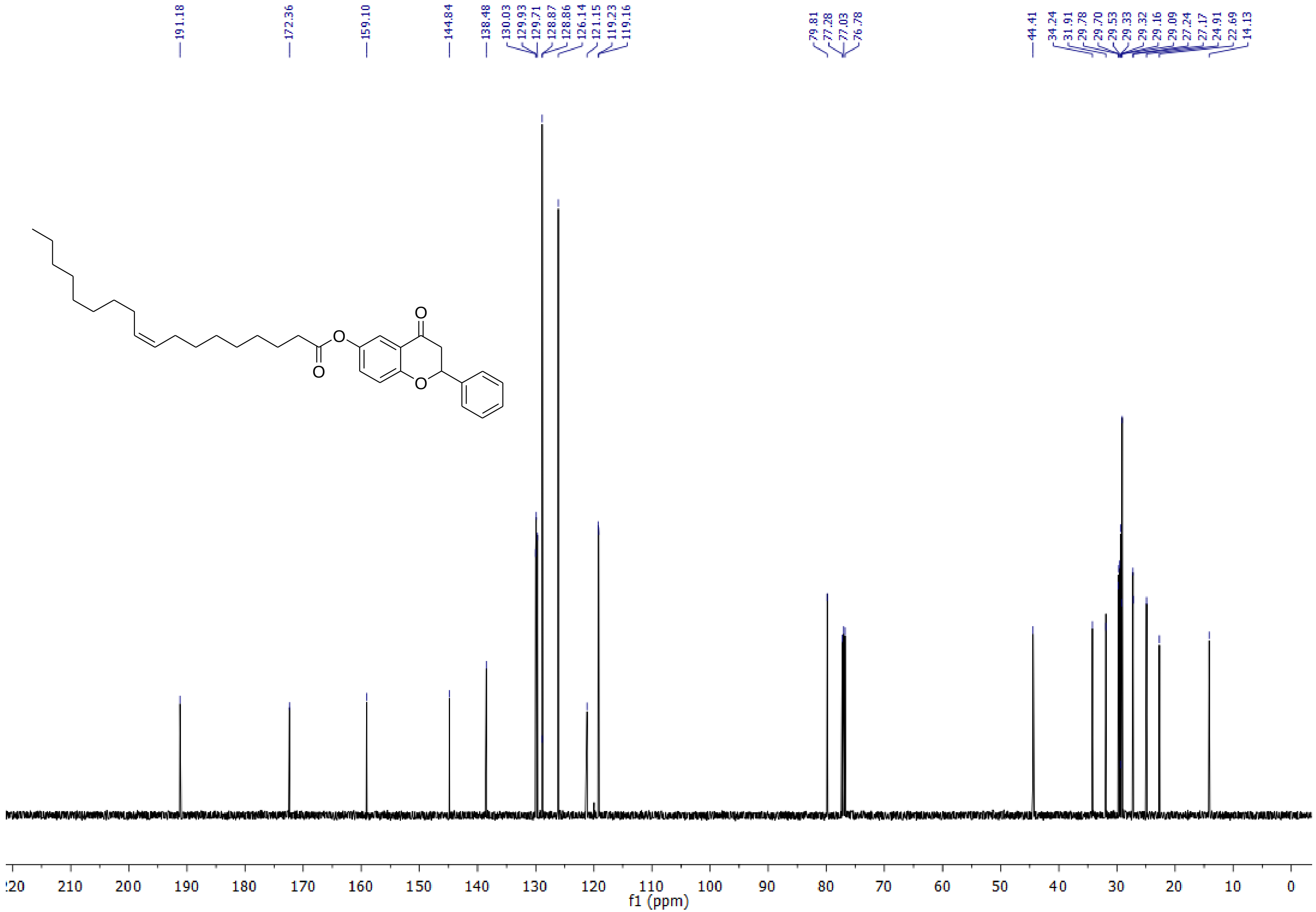
191.18
172.36
159.10
144.84
138.48
130.03
129.93
129.71
128.87
128.86
126.14
121.15
119.23
119.16
79.81
77.28
77.03
76.78
44.41
34.24
31.91
29.78
29.70
29.53
29.33
29.32
29.16
29.09
27.24
27.17
24.91
22.69
14.13
f1 (ppm)

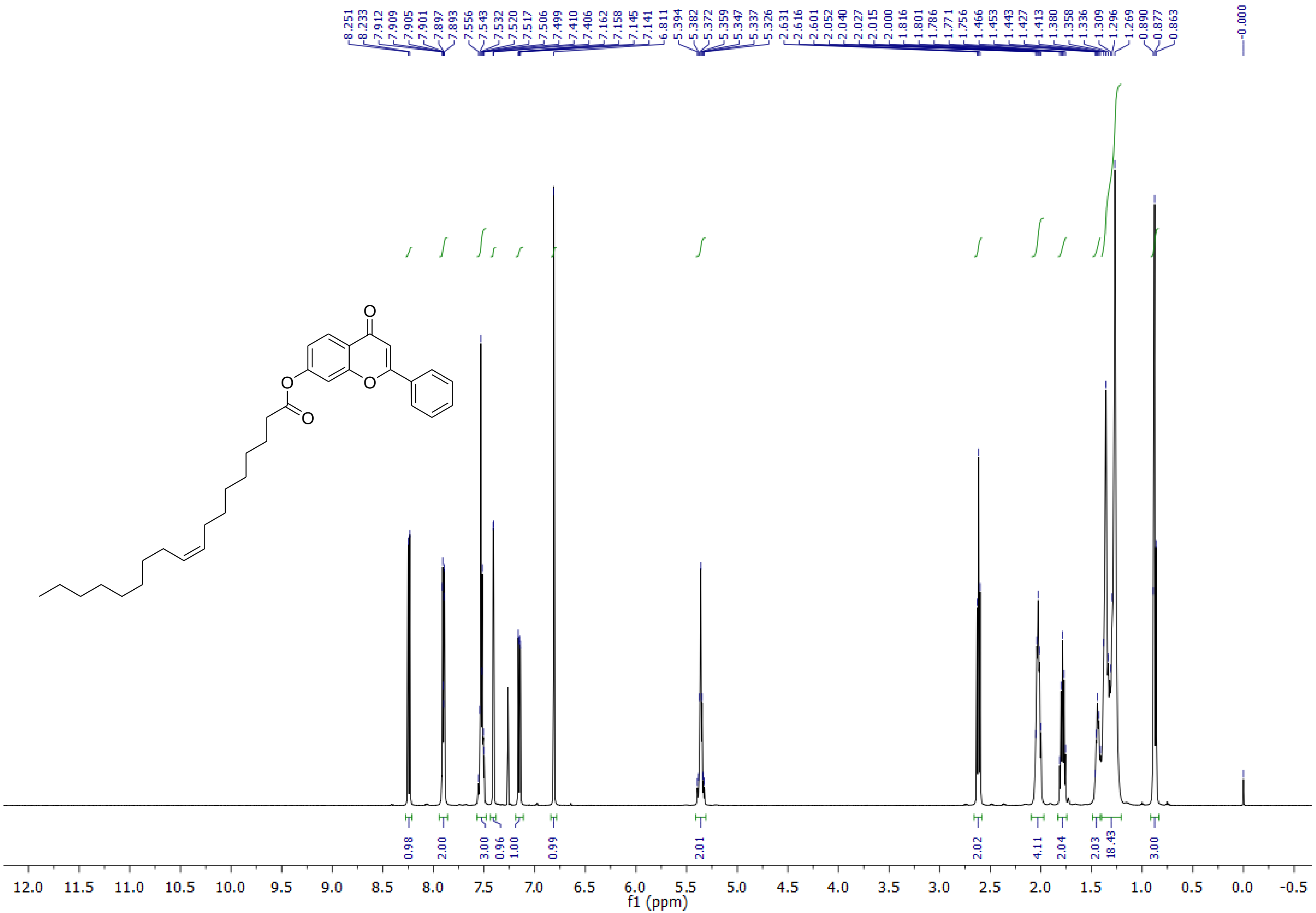

8.251
8.233
7.912
7.909
7.905
7.901
7.897
7.893
7.556
7.543
7.532
7.520
7.517
7.506
7.499
7.410
7.406
7.162
7.158
7.145
7.141
6.811
5.394
5.382
5.372
5.359
5.347
5.337
5.326
2.631
2.616
2.601
2.052
2.040
2.027
2.015
2.000
1.816
1.801
1.786
1.771
1.756
1.466
1.453
1.443
1.427
1.413
1.380
1.358
1.336
1.309
1.296
1.269
0.890
0.877
0.863
0.000
0.98
2.00
3.00
0.96
1.00
0.99
2.01
2.02
4.11
2.04
2.03
18.43
3.00
12.0 11.5 11.0 10.5 10.0 9.5 9.0 8.5 8.0 7.5 7.0 6.5 6.0 5.5 5.0 4.5 4.0 3.5 3.0 2.5 2.0 1.5 1.0 0.5 0.0 -0.5
f1 (ppm)

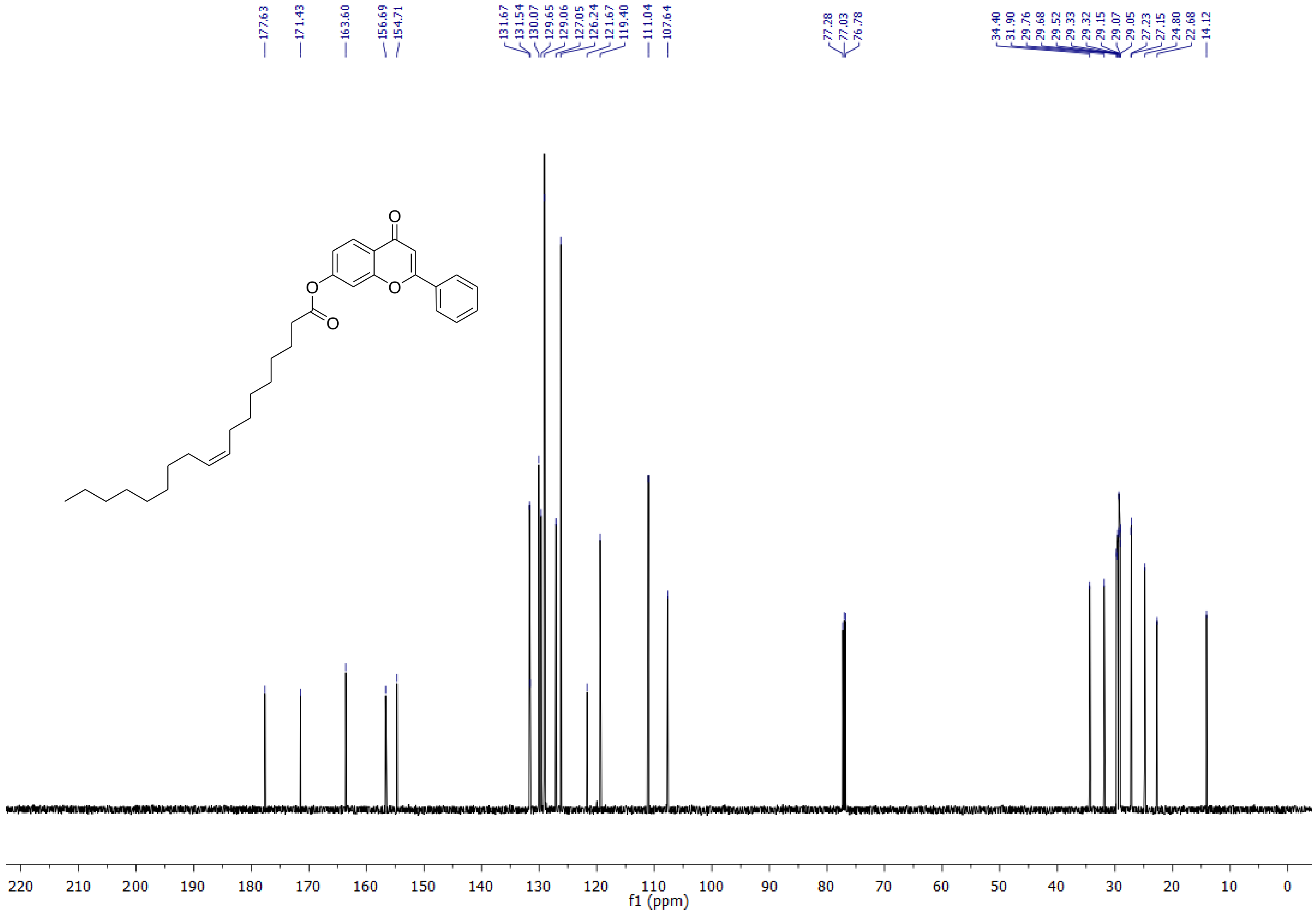
177.63
171.43
163.60
156.69
154.71
131.67
131.54
130.07
129.65
129.06
127.05
126.24
121.67
119.40
111.04
107.64
77.28
77.03
76.78
34.40
31.90
29.76
29.68
29.52
29.33
29.32
29.15
29.07
29.05
27.23
27.15
24.80
22.68
14.12
220 210 200 190 180 170 160 150 140 130 120 110 100 90 80 70 60 50 40 30 20 10 0
f1 (ppm)

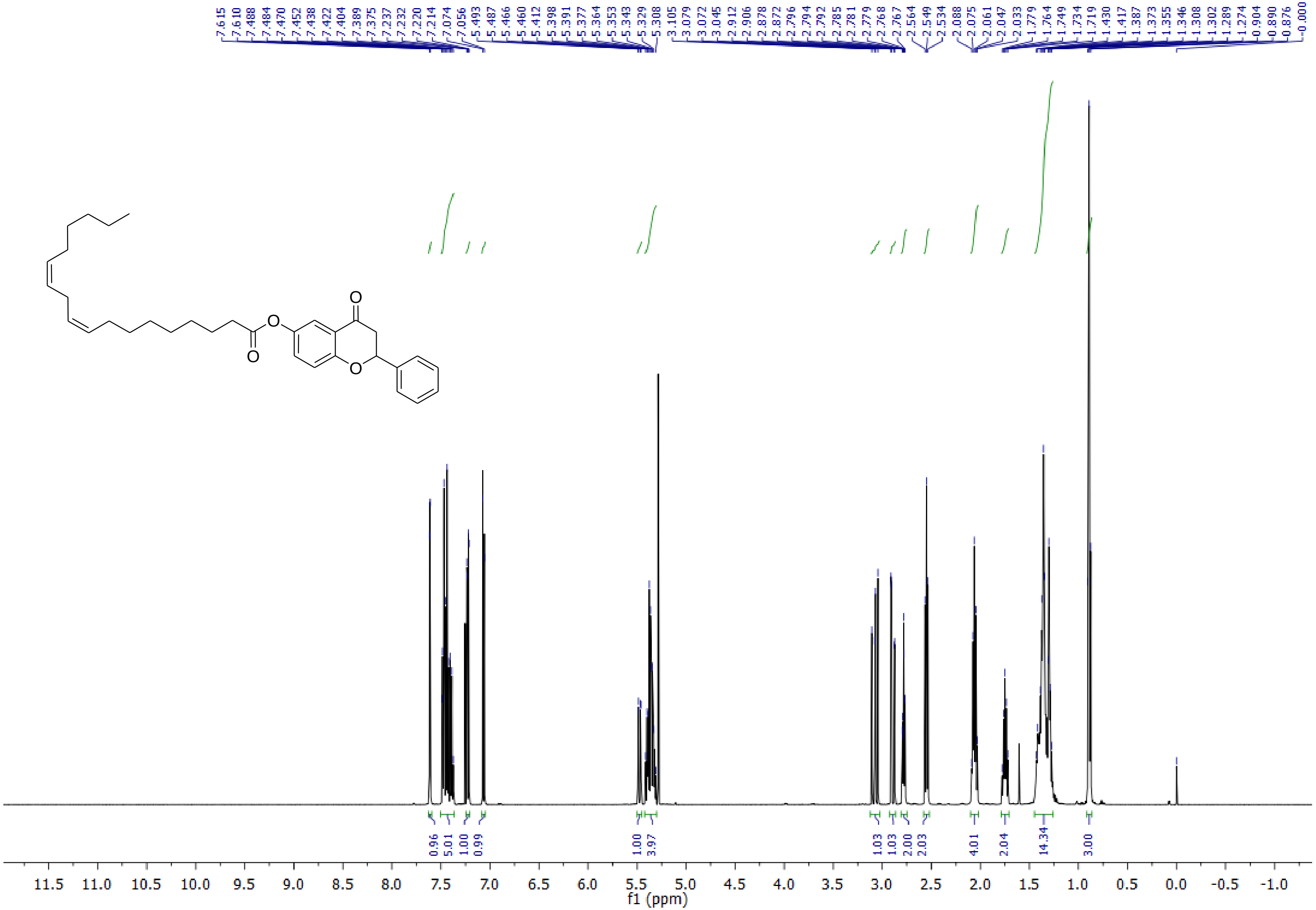
7.615 7.610 7.488 7.484 7.470 7.452 7.438 7.422 7.404 7.389 7.375 7.237 7.232 7.220 7.214 7.074 7.056 5.493 5.487 5.466 5.460 5.412 5.398 5.391 5.377 5.364 5.353 5.343 5.329 5.308 3.105 3.079 3.072 3.045 2.912 2.906 2.878 2.872 2.796 2.794 2.792 2.785 2.781 2.779 2.768 2.767 2.564 2.549 2.534 2.088 2.075 2.061 2.047 2.033 1.779 1.764 1.749 1.734 1.719 1.430 1.417 1.387 1.373 1.355 1.346 1.308 1.302 1.289 1.274 0.904 0.890 0.876 -0.000
O
O
O
O
0.96
5.01
1.00
0.99
1.00
3.97
1.03
1.03
2.00
2.03
4.01
2.04
14.34
3.00
11.5 11.0 10.5 10.0 9.5 9.0 8.5 8.0 7.5 7.0 6.5 6.0 5.5 5.0 4.5 4.0 3.5 3.0 2.5 2.0 1.5 1.0 0.5 0.0 -0.5 -1.0
f1 (ppm)

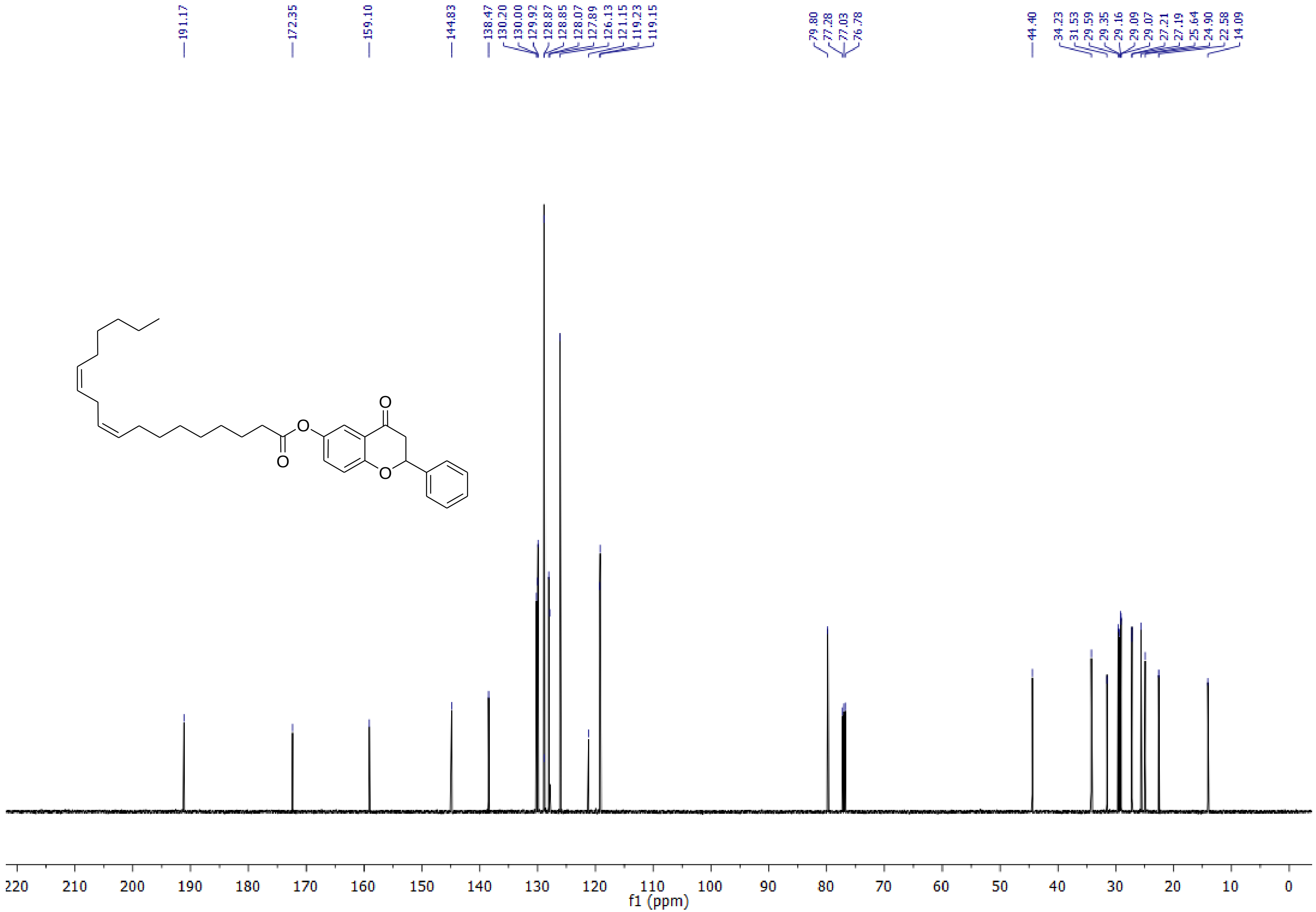

191.17
172.35
159.10
144.83
138.47
130.20
130.00
129.92
128.87
128.85
128.07
127.89
126.13
121.15
119.23
119.15
79.80
77.28
77.03
76.78
44.40
34.23
31.53
29.59
29.35
29.16
29.09
29.07
27.21
27.19
25.64
24.90
22.58
14.09
f1 (ppm)

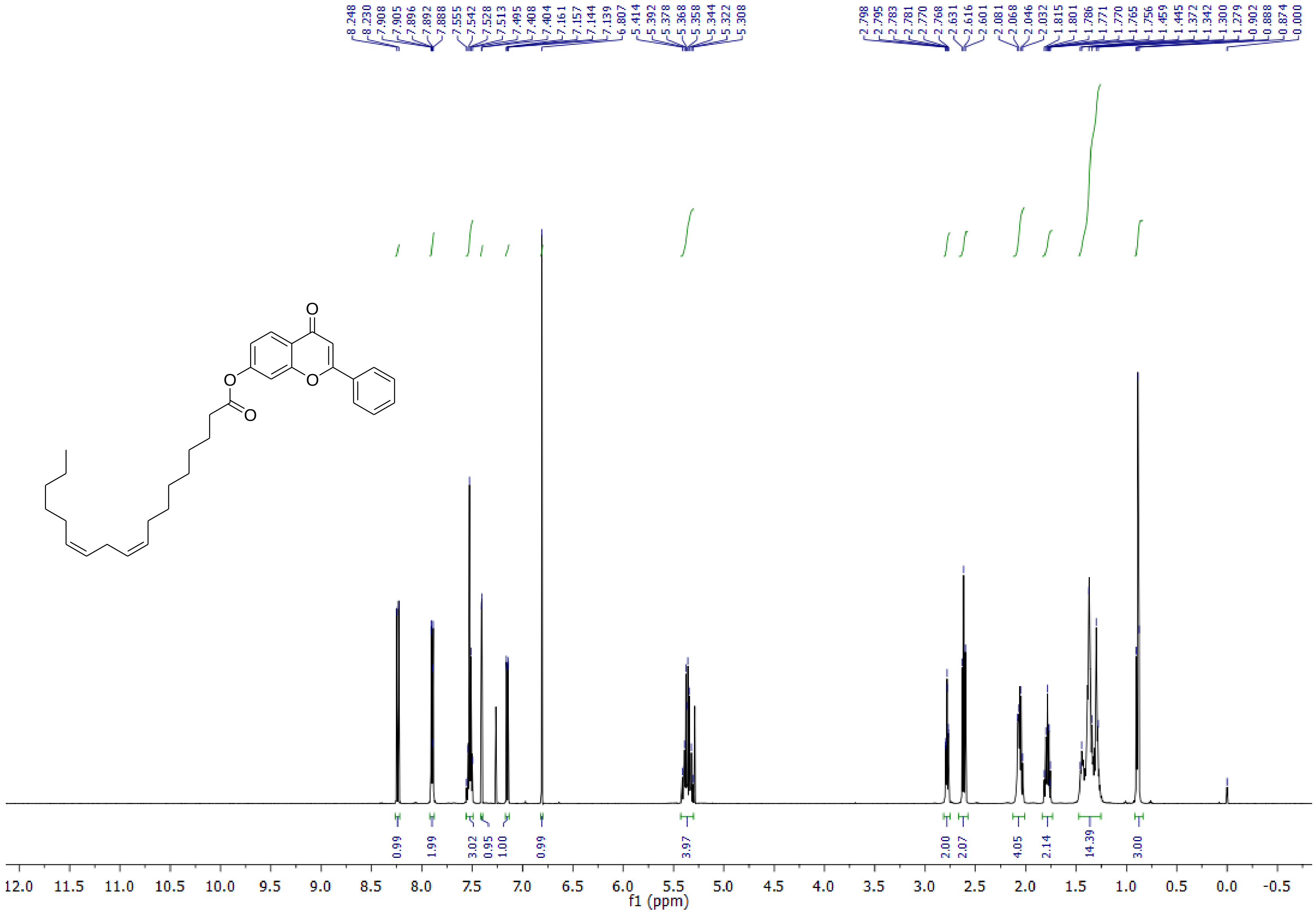
8.248
8.230
7.908
7.905
7.896
7.892
7.888
7.555
7.542
7.528
7.513
7.495
7.408
7.404
7.161
7.157
7.144
7.139
6.807
5.414
5.392
5.378
5.368
5.358
5.344
5.322
5.308
2.798
2.795
2.783
2.781
2.770
2.768
2.631
2.616
2.601
2.081
2.068
2.046
2.032
1.815
1.801
1.786
1.771
1.770
1.765
1.756
1.459
1.445
1.372
1.342
1.300
1.279
0.902
0.888
0.874
0.000
0.99
1.99
3.02
0.95
1.00
0.99
3.97
2.00
2.07
4.05
2.14
14.39
3.00
12.0 11.5 11.0 10.5 10.0 9.5 9.0 8.5 8.0 7.5 7.0 6.5 6.0 5.5 5.0 4.5 4.0 3.5 3.0 2.5 2.0 1.5 1.0 0.5 0.0 -0.5
f1 (ppm)

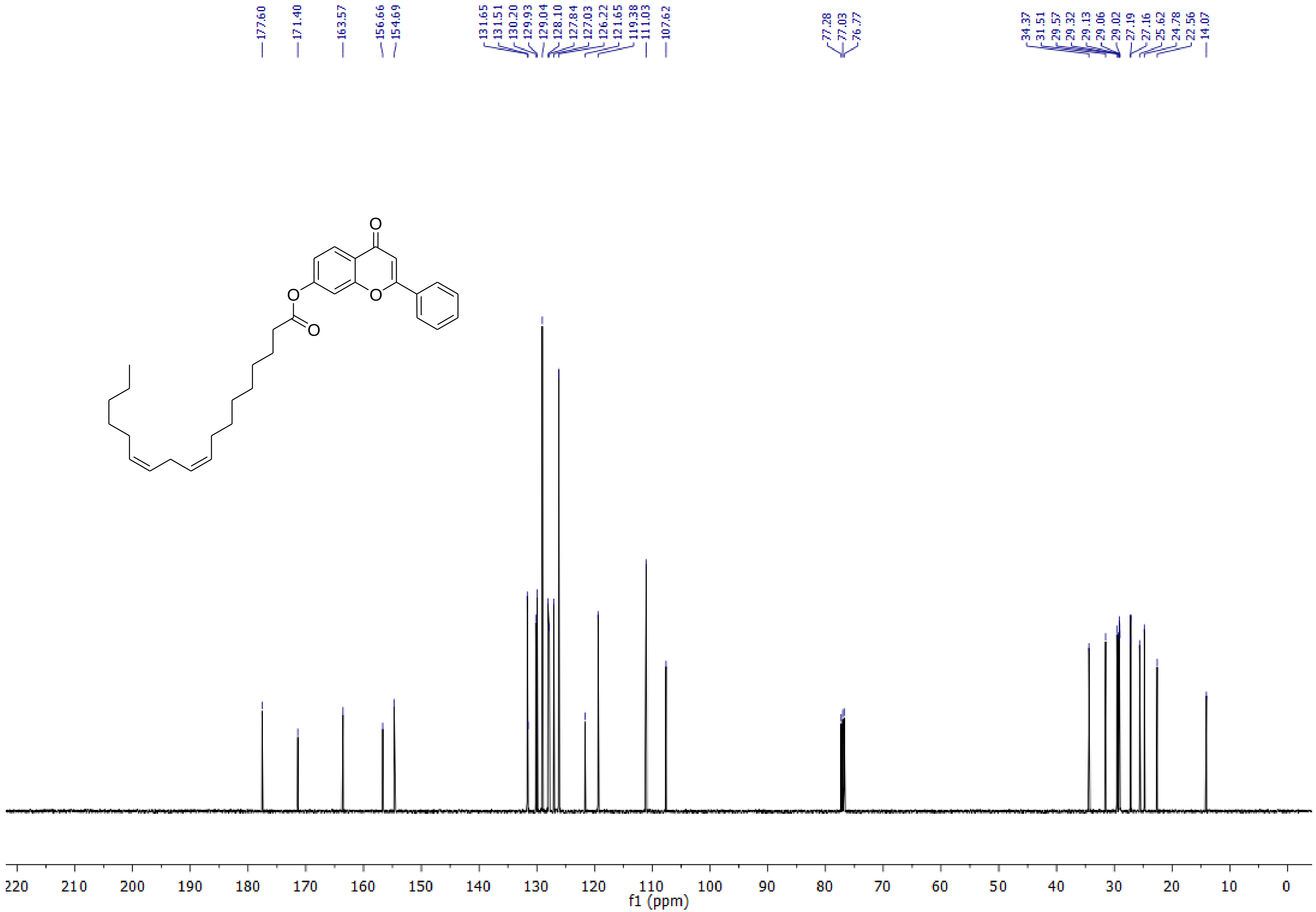

177.60
171.40
163.57
156.66
154.69
131.65
131.51
130.20
129.93
129.04
128.10
127.84
127.03
126.22
121.65
119.38
111.03
107.62
77.28
77.03
76.77
34.37
31.51
29.57
29.32
29.13
29.06
29.02
27.19
27.16
25.62
24.78
22.56
14.07
O
O
O
O
220 210 200 190 180 170 160 150 140 130 120 110 100 90 80 70 60 50 40 30 20 10 0
f1 (ppm)

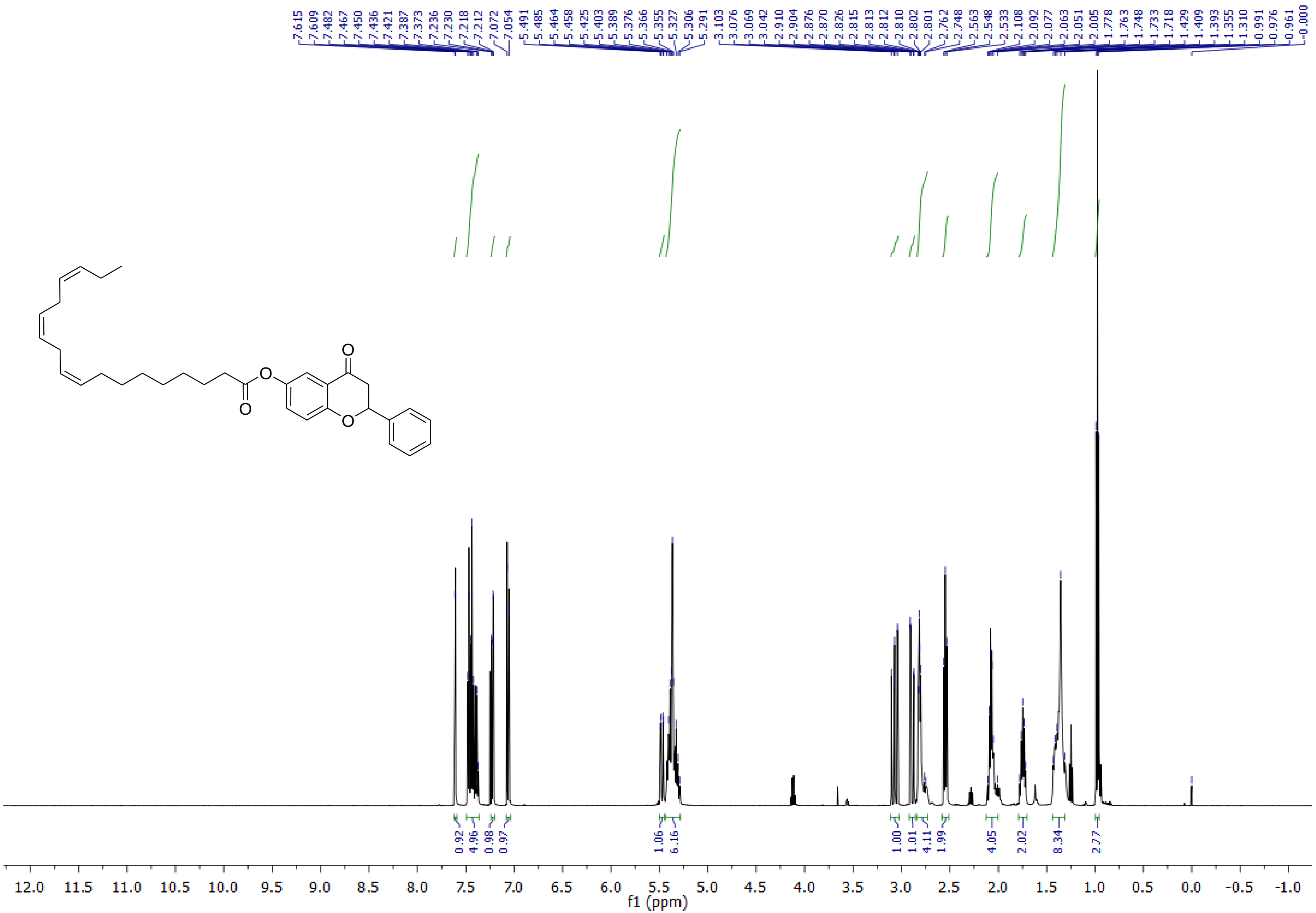
7.615 7.609 7.482 7.467 7.450 7.436 7.421 7.387 7.373 7.236 7.230 7.218 7.212 7.072 7.054
5.491 5.485 5.464 5.458 5.425 5.403 5.389 5.376 5.366 5.355 5.327 5.306 5.291
3.103 3.076 3.069 3.042 2.910 2.904 2.876 2.870 2.826 2.815 2.813 2.812 2.810 2.802 2.801 2.762 2.748 2.563 2.548 2.533
2.108 2.092 2.077 2.063 2.051 2.005 1.778 1.763 1.748 1.733 1.718 1.429 1.409 1.393 1.355 1.310 0.991 0.976 0.961 -0.000
O
O
O
O
O
0.92 4.96 0.98 0.97 1.06 6.16 1.00 1.01 4.11 1.99 4.05 2.02 8.34 2.77
12.0 11.5 11.0 10.5 10.0 9.5 9.0 8.5 8.0 7.5 7.0 6.5 6.0 5.5 5.0 4.5 4.0 3.5 3.0 2.5 2.0 1.5 1.0 0.5 0.0 -0.5 -1.0
f1 (ppm)

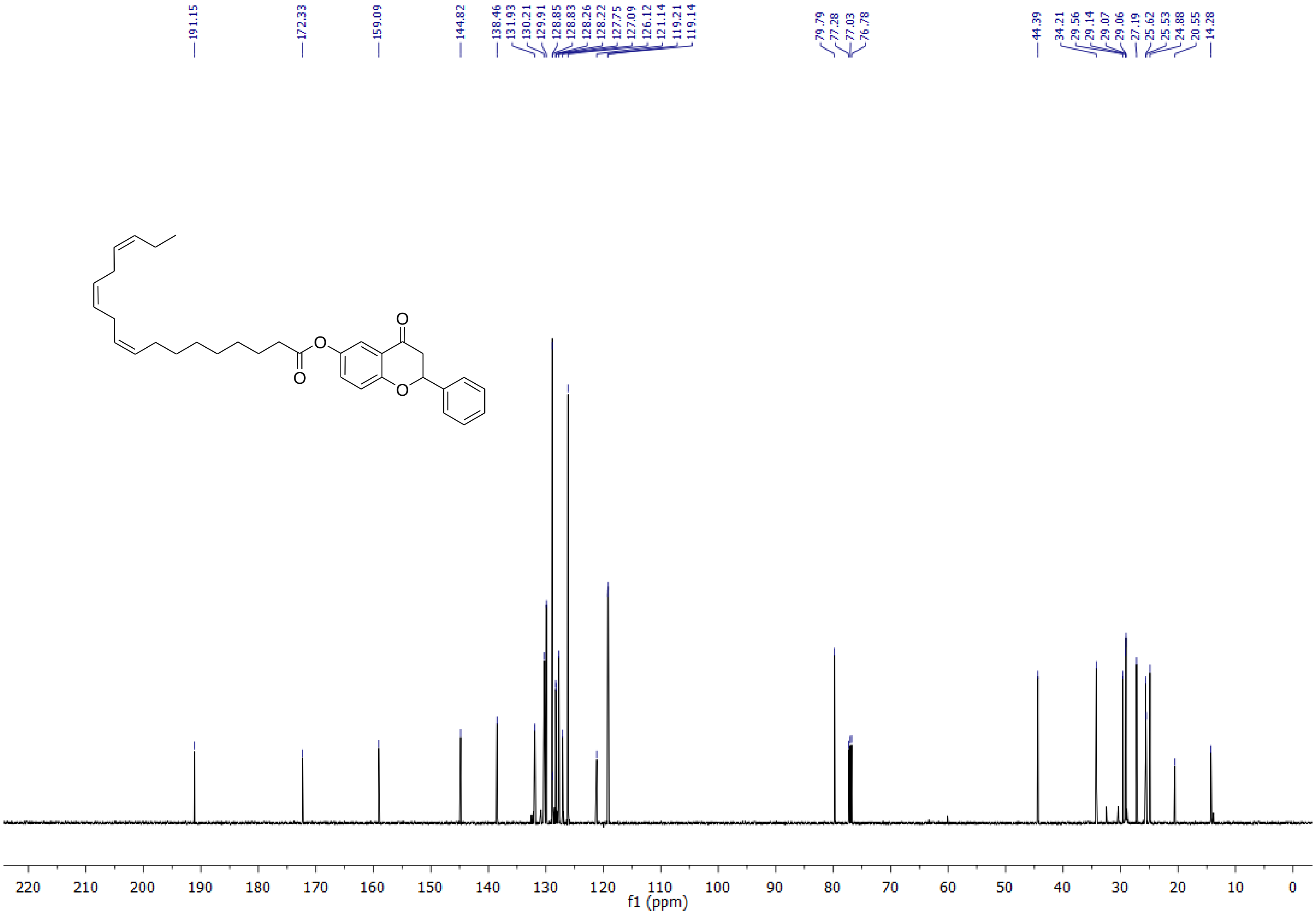
191.15
172.33
159.09
144.82
138.46
131.93
130.21
129.91
128.85
128.83
128.26
128.22
127.75
127.09
126.12
121.14
119.21
119.14
79.79
77.28
77.03
76.78
44.39
34.21
29.56
29.14
29.07
29.06
27.19
25.62
25.53
24.88
20.55
14.28
220 210 200 190 180 170 160 150 140 130 120 110 100 90 80 70 60 50 40 30 20 10 0
f1 (ppm)

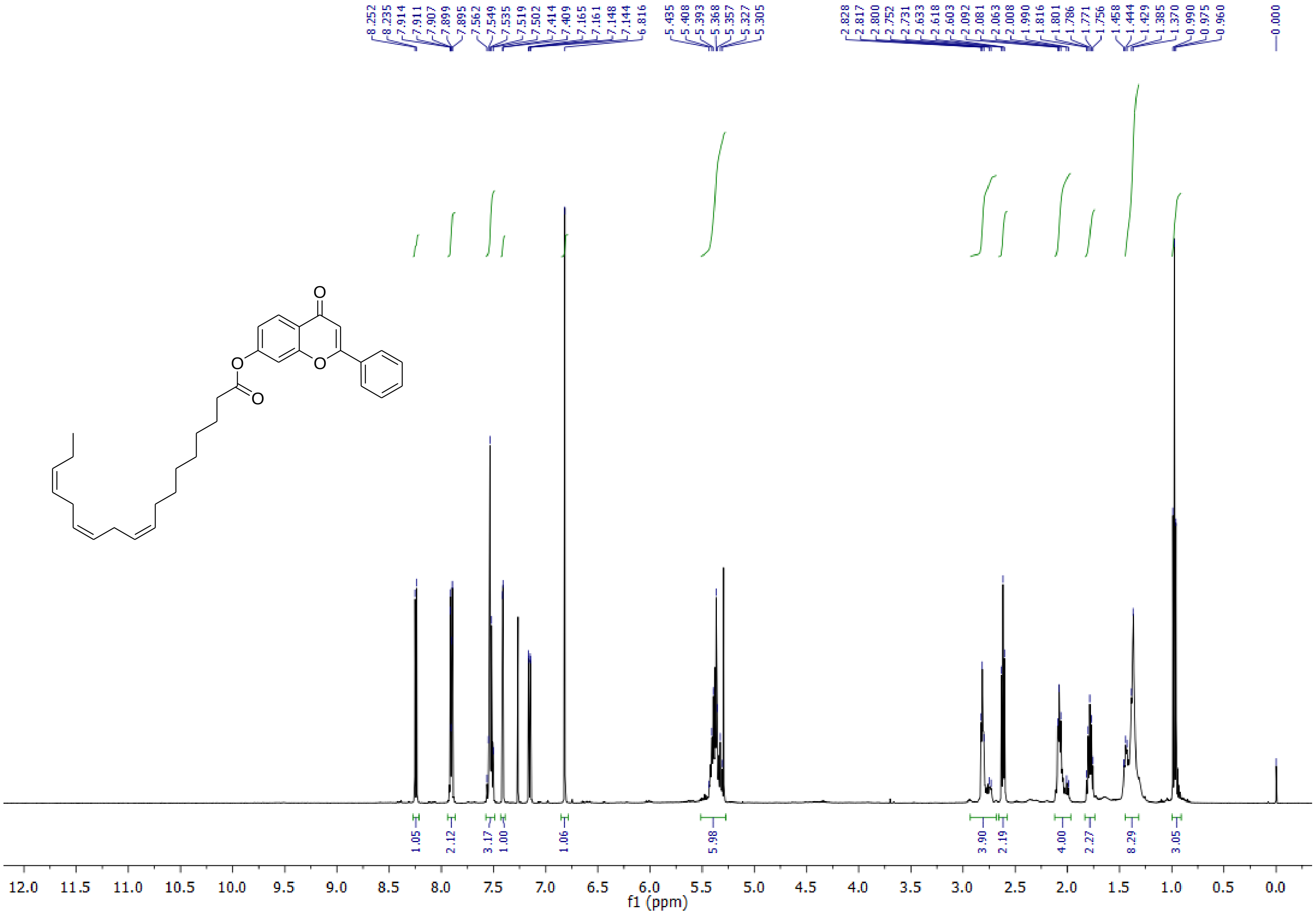
8.252
8.235
7.914
7.911
7.907
7.899
7.895
7.562
7.549
7.535
7.519
7.502
7.414
7.409
7.165
7.161
7.148
7.144
6.816
5.435
5.408
5.393
5.368
5.357
5.327
5.305
2.828
2.817
2.800
2.752
2.731
2.633
2.618
2.603
2.092
2.081
2.063
2.008
1.990
1.816
1.801
1.786
1.771
1.756
1.458
1.444
1.429
1.385
1.370
0.990
0.975
0.960
0.000
1.05
2.12
3.17
1.00
1.06
5.98
3.90
2.19
4.00
2.27
8.29
3.05
12.0
11.5
11.0
10.5
10.0
9.5
9.0
8.5
8.0
7.5
7.0
6.5
6.0
5.5
5.0
4.5
4.0
3.5
3.0
2.5
2.0
1.5
1.0
0.5
0.0
f1 (ppm)

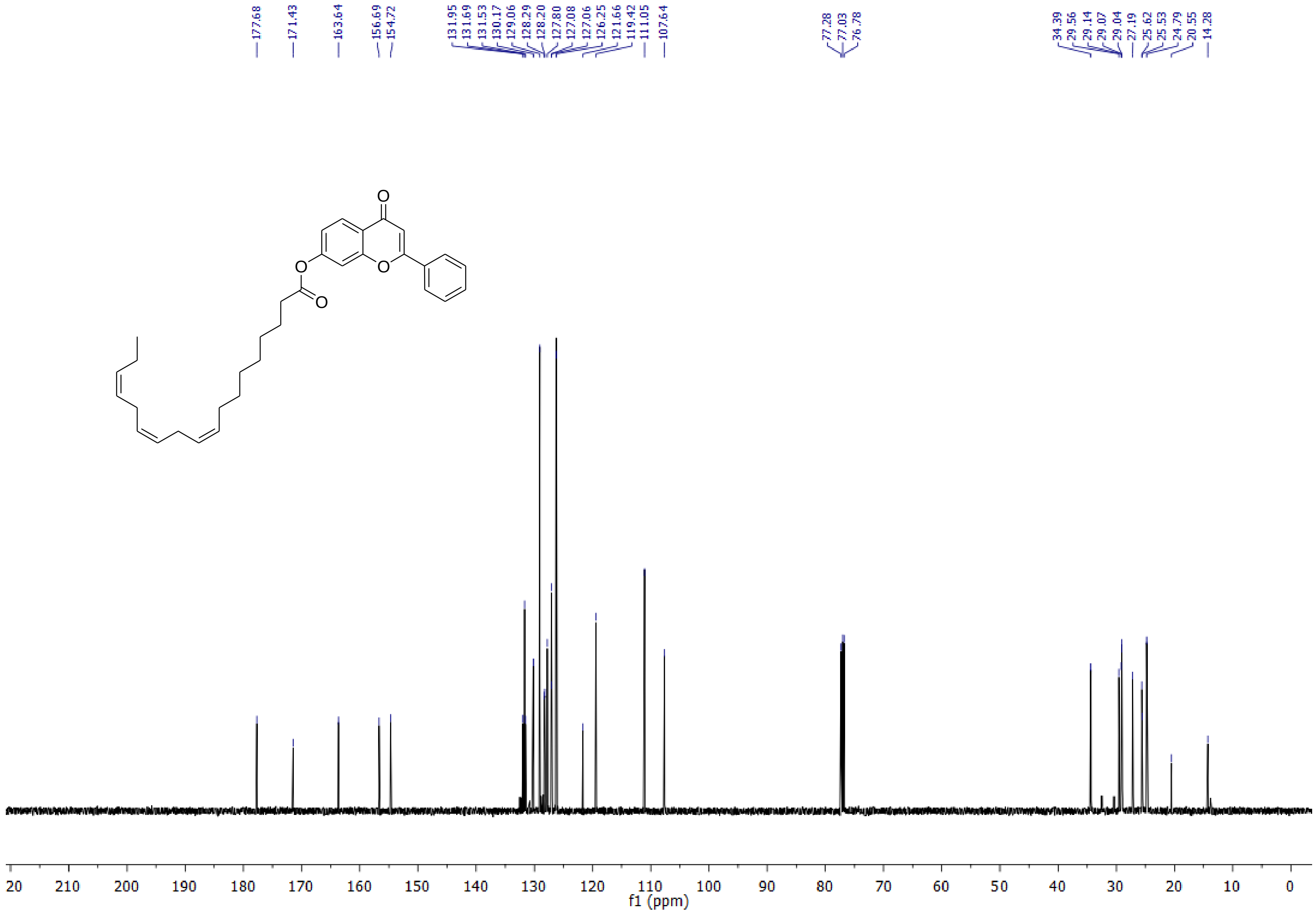

177.68
171.43
163.64
156.69
154.72
131.95
131.69
131.53
130.17
129.06
128.29
128.20
127.80
127.08
127.06
126.25
121.66
119.42
111.05
107.64
77.28
77.03
76.78
34.39
29.56
29.14
29.07
29.04
27.19
25.62
25.53
24.79
20.55
14.28
O
O
O
O
f1 (ppm)

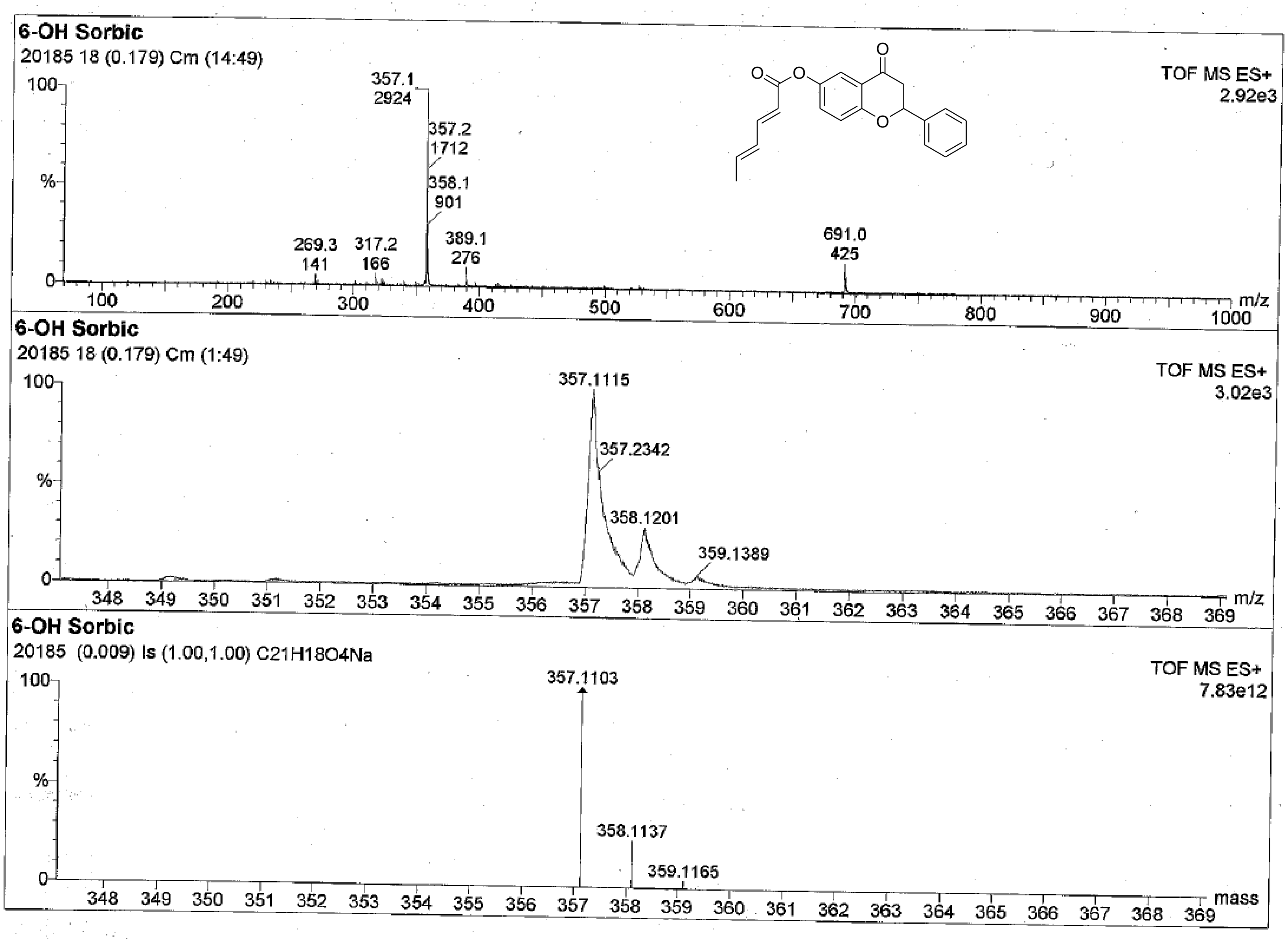

6-OH Sorbic
20185 18 (0.179) Cm (14:49)
TOF MS ES+
2.92e3
357.1
2924
357.2
1712
358.1
901
269.3
141
317.2
166
389.1
276
691.0
425
m/z
6-OH Sorbic
20185 18 (0.179) Cm (1:49)
TOF MS ES+
3.02e3
357.1115
357.2342
358.1201
359.1389
m/z
6-OH Sorbic
20185 (0.009) Is (1.00,1.00) C21H18O4Na
TOF MS ES+
7.83e12
357.1103
358.1137
359.1165
mass

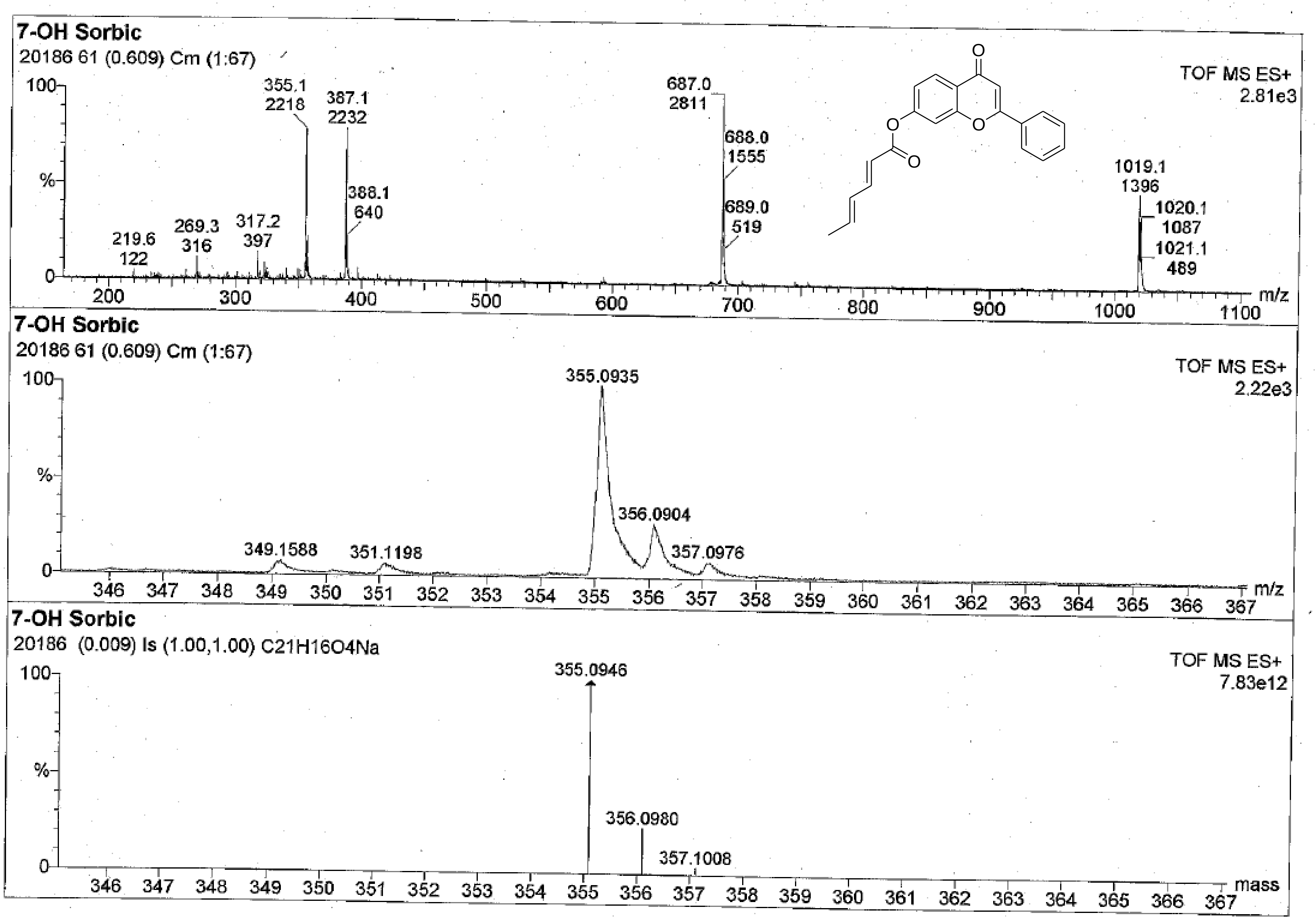
7-OH Sorbic
20186 61 (0.609) Cm (1:67)
TOF MS ES+
2.81e3
355.1
2218
387.1
2232
388.1
640
219.6
122
269.3
316
317.2
397
687.0
2811
688.0
1555
689.0
519
1019.1
1396
1020.1
1087
1021.1
489
m/z
7-OH Sorbic
20186 61 (0.609) Cm (1:67)
TOF MS ES+
2.22e3
355.0935
356.0904
357.0976
349.1588
351.1198
m/z
7-OH Sorbic
20186 (0.009) Is (1.00,1.00) C21H16O4Na
TOF MS ES+
7.83e12
355.0946
356.0980
357.1008
mass

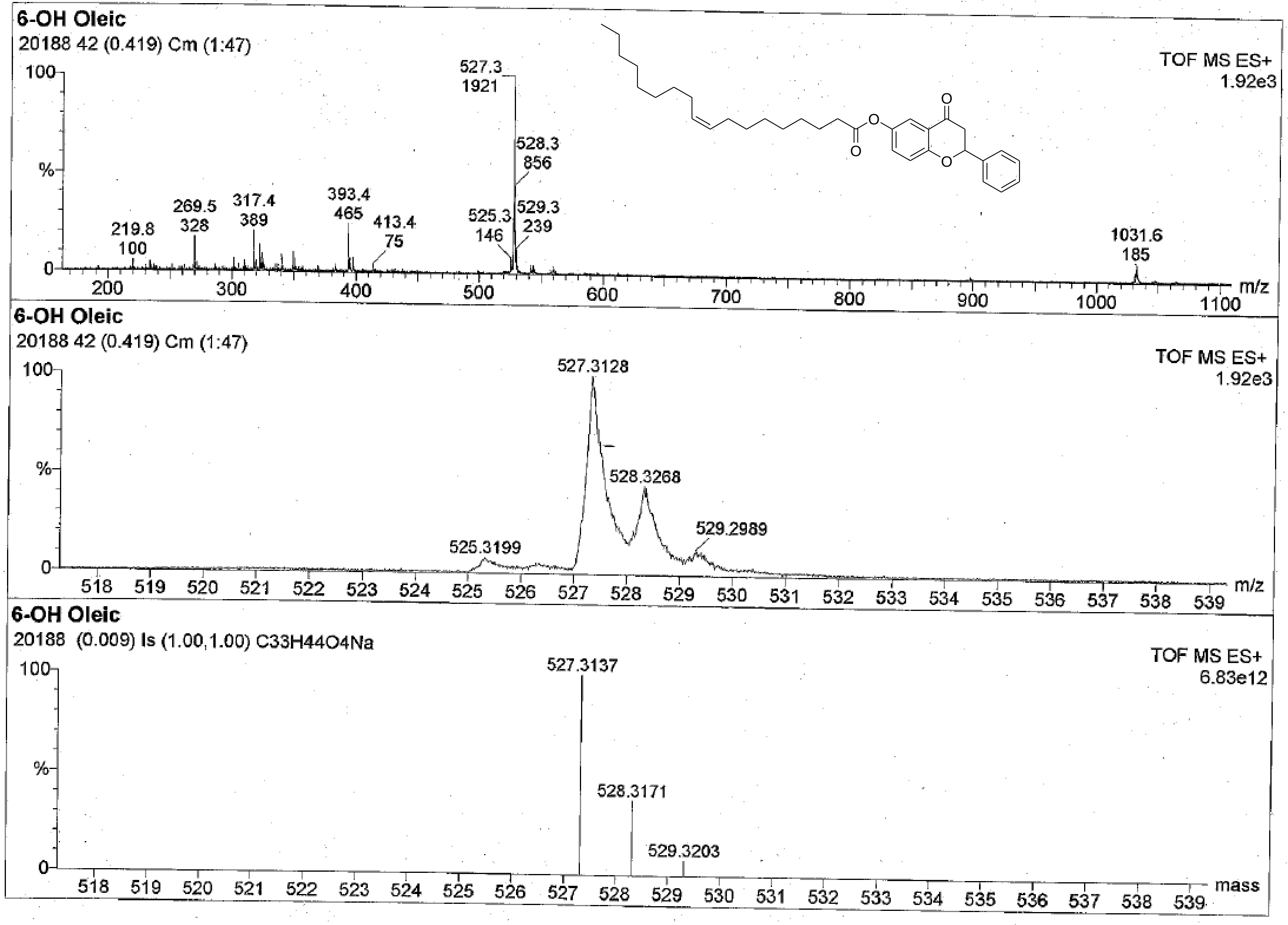
6-OH Oleic
20188 42 (0.419) Cm (1:47)
TOF MS ES+
1.92e3
527.3
1921
528.3
856
529.3
239
525.3
146
393.4
465
413.4
75
317.4
389
269.5
328
219.8
100
1031.6
185
m/z
6-OH Oleic
20188 42 (0.419) Cm (1:47)
TOF MS ES+
1.92e3
527.3128
528.3268
529.2989
525.3199
m/z
6-OH Oleic
20188 (0.009) Is (1.00,1.00) C33H44O4Na
TOF MS ES+
6.83e12
527.3137
528.3171
529.3203
mass

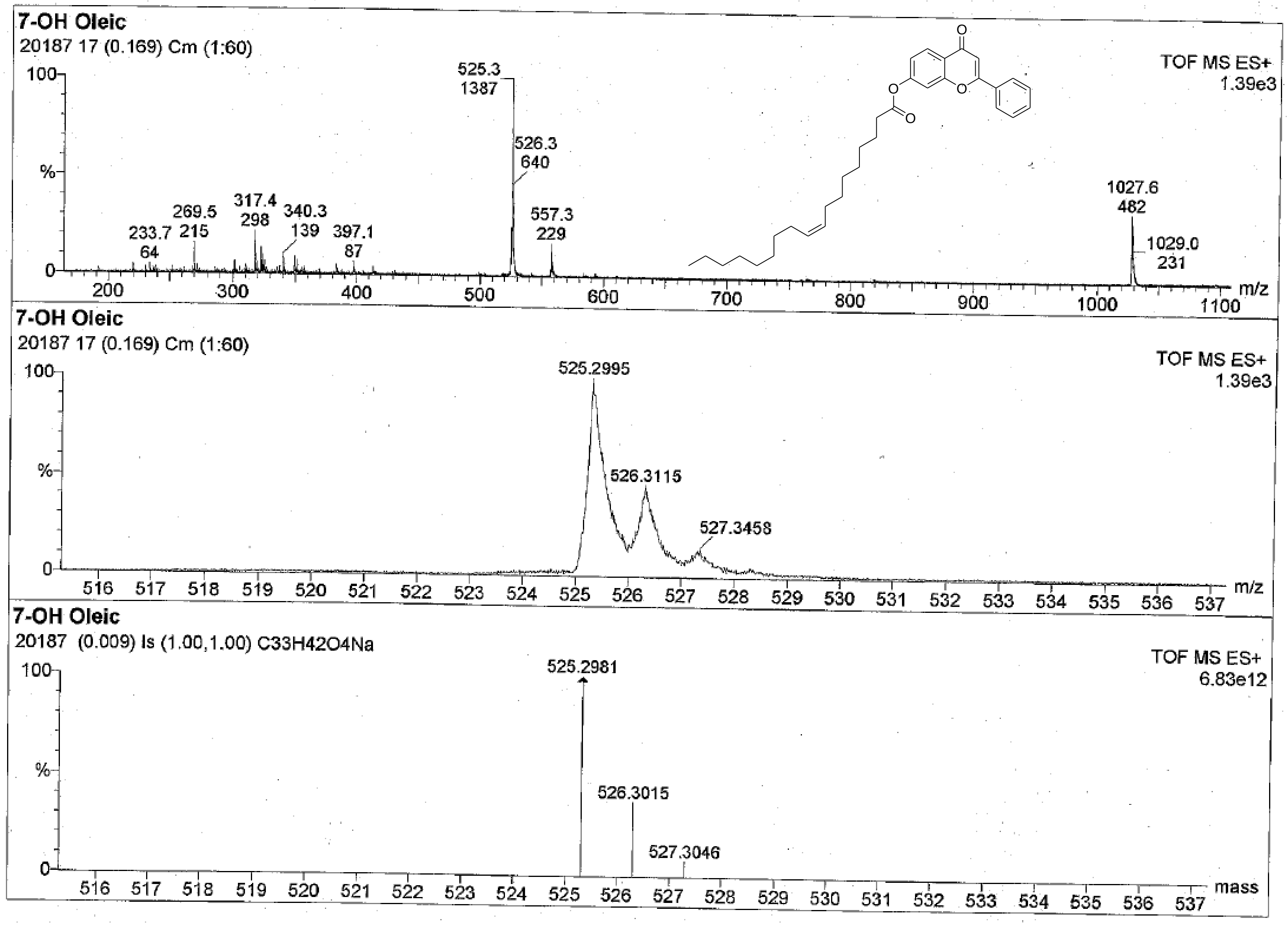

7-OH Oleic
20187 17 (0.169) Cm (1:60)
TOF MS ES+
1.39e3
525.3
1387
526.3
640
557.3
229
269.5
215
233.7
64
317.4
298
340.3
139
397.1
87
1027.6
482
1029.0
231
m/z
7-OH Oleic
20187 17 (0.169) Cm (1:60)
TOF MS ES+
1.39e3
525.2995
526.3115
527.3458
m/z
7-OH Oleic
20187 (0.009) Is (1.00,1.00) C33H42O4Na
TOF MS ES+
6.83e12
525.2981
526.3015
527.3046
mass
